# Vinculin Regulates the Recruitment and Release of Core Focal Adhesion Proteins in a Force-Dependent Manner

**DOI:** 10.1016/j.cub.2013.01.009

**Published:** 2013-02-18

**Authors:** Alex Carisey, Ricky Tsang, Alexandra M. Greiner, Nadja Nijenhuis, Nikki Heath, Alicja Nazgiewicz, Ralf Kemkemer, Brian Derby, Joachim Spatz, Christoph Ballestrem

**Affiliations:** 1 Wellcome Trust Centre for Cell-Matrix Research, Faculty of Life Sciences, University of Manchester, Oxford Road, Manchester M13 9PT, England, UK; 2 Department of New Materials and Biosystems, Max Planck Institute for Metals Research, Heisenbergstrasse 3, 70569 Stuttgart, Germany; 3 School of Materials, University of Manchester, Oxford Road, Manchester M13 9PL, England, UK; 4 Department of Biophysical Chemistry, University of Heidelberg, 69120 Heidelberg, Germany

## Abstract

**Background:**

Cells sense the extracellular environment using adhesion receptors (integrins) linked to the intracellular actin cytoskeleton through a complex network of regulatory proteins that, all together, form focal adhesions (FAs). The molecular basis of how these sensing units are regulated, how they are implicated in transducing mechanical stimuli, and how this leads to a spatiotemporal coordination of FAs is unclear.

**Results:**

Here we show that vinculin, through its links to the talin-integrin complex and F-actin, regulates the transmission of mechanical signals from the extracellular matrix to the actomyosin machinery. We demonstrate that the vinculin interaction with the talin-integrin complex drives the recruitment and release of core FA components. The activation state of vinculin is itself regulated by force, as underscored by our observation that vinculin localization to FAs is dependent on actomyosin contraction. Using a variety of vinculin mutants, we establish which components of the cell-matrix adhesion network are coordinated through direct and indirect associations with vinculin. Moreover, using cyclic stretching, we demonstrate that vinculin plays a key role in the transmission of extracellular mechanical stimuli leading to the reorganization of cell polarity. Of particular importance is the actin-binding tail region of vinculin, without which the cell’s ability to repolarize in response to cyclic stretching is perturbed.

**Conclusions:**

Overall our data promote a model whereby vinculin controls the transmission of intracellular and extracellular mechanical cues that are important for the spatiotemporal assembly, disassembly, and reorganization of FAs to coordinate polarized cell motility.

## Highlights

► Intracellular tension is required to maintain vinculin in focal adhesions ► Vinculin activity stabilizes the talin-integrin complex and increases cell adhesion ► Vinculin coordinates the release of FA proteins through the talin-integrin complex ► Vinculin coordinates stretch-induced cell polarization via its link to actin

## Introduction

The ability of cells to communicate with their environment is essential for all developmental and physiological processes. Cells sense the chemical and mechanical properties of their environment through cell-matrix adhesion sites known as FAs. In FAs, integrins, which are the main adhesion receptors binding to extracellular matrix proteins, are linked to the actin cytoskeleton by a large number of FA plaque proteins [1, 2]. The appearance of FAs is dependent on the tension exerted by the contractile actomyosin machinery [3, 4]. Inhibition of pathways that lead to myosin II activation results in the disassembly of adhesion clusters [5, 6], indicating that tensile forces contribute to the stability of FAs. However, the way that cells sense and transmit forces that lead to the reorganization of these structures is not clear.

Vinculin is one of the core FA proteins appearing in the early stages of FA formation in small dot-like adhesion complexes at the cell periphery that mature into larger streak-like FAs [7]. The presence of talin is required for vinculin recruitment to FAs [8, 9], and paxillin may contribute to this [10]. Through interactions with the talin-integrin complex and the actin cytoskeleton [11–13], vinculin is ideally positioned to coordinate force-induced signals. The hypothesis that vinculin is part of the force machinery regulating FAs derives from the observations that its recruitment to FAs correlates with subcellular areas of increased tensile forces in cells [14] and that tensile forces act on vinculin itself [15]. However, the precise function of vinculin as a force-transducing protein remains unclear.

Structurally, vinculin consists of a headpiece and a tail region separated by a flexible proline-rich neck region [12]. It can adopt either an inactive, globular conformation or an active, extended conformation. In the inactive state, a head-tail interaction masks binding sites [16]. The activated form of vinculin primarily localizes to FAs [17] where the binding sites for its many partners, including talin and α-actinin (which bind to the head domain), VASP, vinexins, ponsin, and Arp2/3 (which bind to the neck region), and paxillin, F-actin, and PIP_2_ (which bind to the tail domain) are exposed [18]. The interaction with talin, the main regulator of integrin activation [19], is essential for the role of vinculin in FA stabilization [20], whereas binding of vinculin to F-actin contributes to the ability of the cell to exert tensile forces on the extracellular matrix (ECM). Binding of both actin and talin together is hypothesized to be necessary for the full activation of vinculin [21, 22].

In this study, we test the hypothesis that vinculin coordinates force-mediated signals. We used a combination of techniques, including atomic force spectroscopy and a variety of imaging methods, to show how vinculin coordinates core FA proteins that regulate polarized cell migration. In addition, we show that vinculin, via its actin binding tail, is involved in the transmission of mechanical stimuli, which is essential for cellular responses to cyclic stretching forces.

## Results

### Vinculin Activity Governs the Recruitment and Release of FA Components

**Figure 1:**
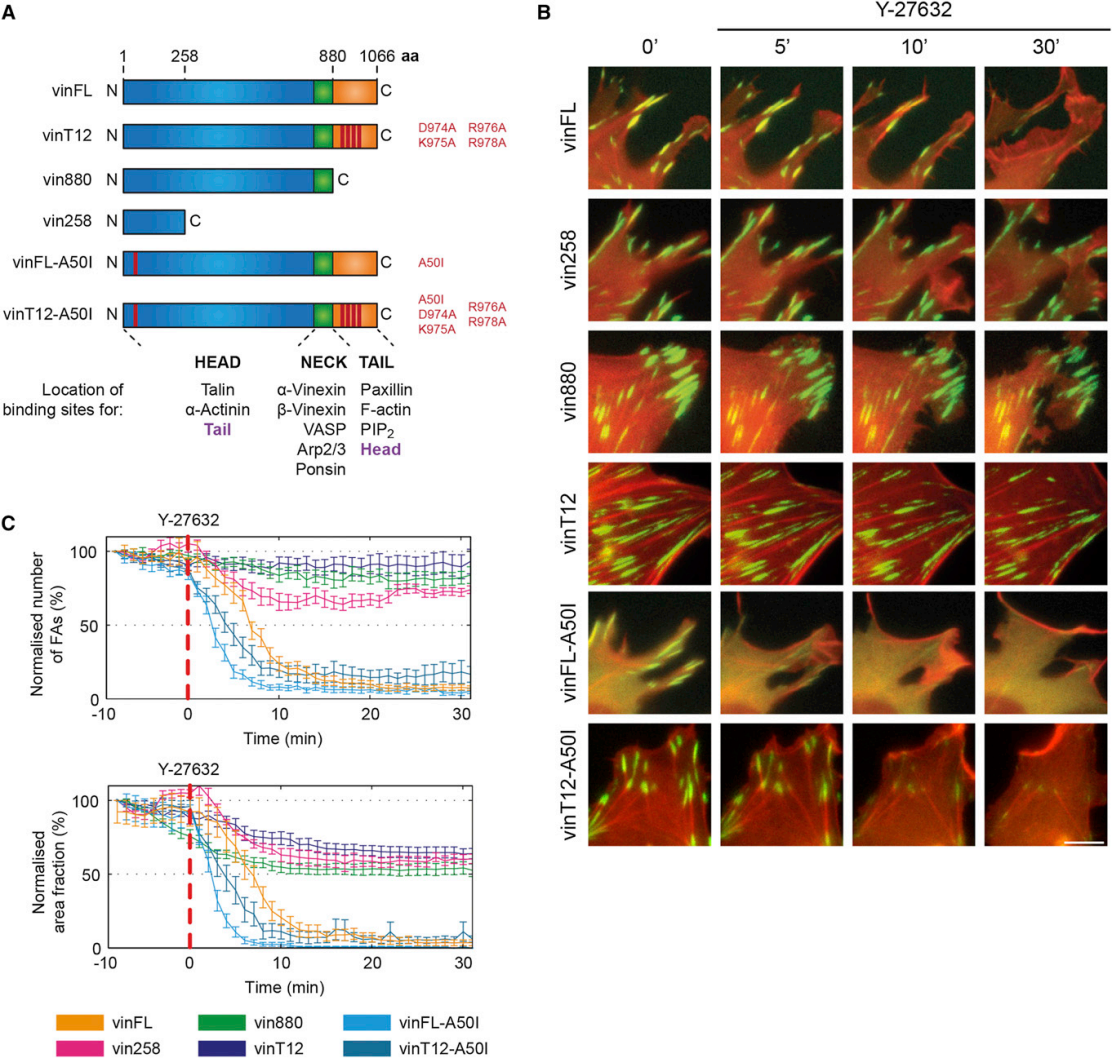
Talin Binding of Active Vinculin Is Essential for Its Ability to Bypass the Requirement of Actomyosin Tension for FA Stabilization (A) Graphical representation of vinculin constructs that were expressed as GFP-fusion constructs in this study. Proteins binding to the individual domains of vinculin are indicated below. (B) *MEFvin*^*−/−*^ cells coexpressing LifeAct-mRFP together with indicated vinculin constructs were treated with Y-27632. Full-length vinculin, but not vin258, vin880, and vinT12 (Movie S1), leaves FAs under these conditions. Vinculin forms carrying an A50I mutation that reduces their binding to talin (vinFL-A50I and vinT12-A50I) are released from FAs in the absence of intracellular tension. Similar results were obtained by treating the cells with cytochalasin D and blebbistatin (Figure S1 and Movie S1). Scale bar represents 5 μm. (C) Quantification of the normalized number and area fraction of vinculin-positive FAs over time. The vertical red dashed line indicates the beginning of the Y-27632 treatment (±SEM, n > 10 cells from two independent experiments).

In previous experiments, we have shown that vin880, a vinculin form without its C-terminal actin-binding tail (Figure 1A), stabilizes FAs such that they become resistant to tension-releasing drugs [20]. To extend these studies, we treated vinculin-deficient (*MEFvin*^−*/−*^) cells expressing just the vinculin D1 domain (vin258), constitutively active full-length vinculin (vinT12), or vinculin wild-type (vinFL) with the Rho-kinase (ROCK) inhibitor Y-27632, the myosin II inhibitor blebbistatin, or the actin-disrupting reagent cytochalasin D. All treatments resulted in a rapid release of full-length vinculin, but not of vin258, vin880, or vinT12 from FAs (Figures 1B and 1C; see also Figure S1 and Movie S1 available online). Analysis of a large number of cells showed that FA stabilization by vin258, vin880, and vinT12 was achieved at low expression levels (data not shown). Interestingly, adding blebbistatin or Y-27632 to cells transfected with vin880 before spreading had a similar effect to adding these substances after cell spreading. In both cases, cells were able to form new adhesion complexes at the cell front (Figure S1C), indicating the potential of preactivated vinculin in triggering FA formation through inside-out signals.

To test whether vinculin binding to talin is critical for the stabilization of FAs under tension-releasing conditions, we analyzed cells expressing constitutively active vinculin bearing an additional A50I mutation, which dramatically reduces talin binding [21]. Interestingly, vinT12-A50I quickly disappeared from FAs upon treatment with tension-releasing drugs (Figures 1B, 1C, S1A, and S1B, and Movie S1), with similar kinetics to vinFL-A50I, indicating that strong binding to talin is essential for vinculin-mediated stabilization of FAs.

**Figure 2:**
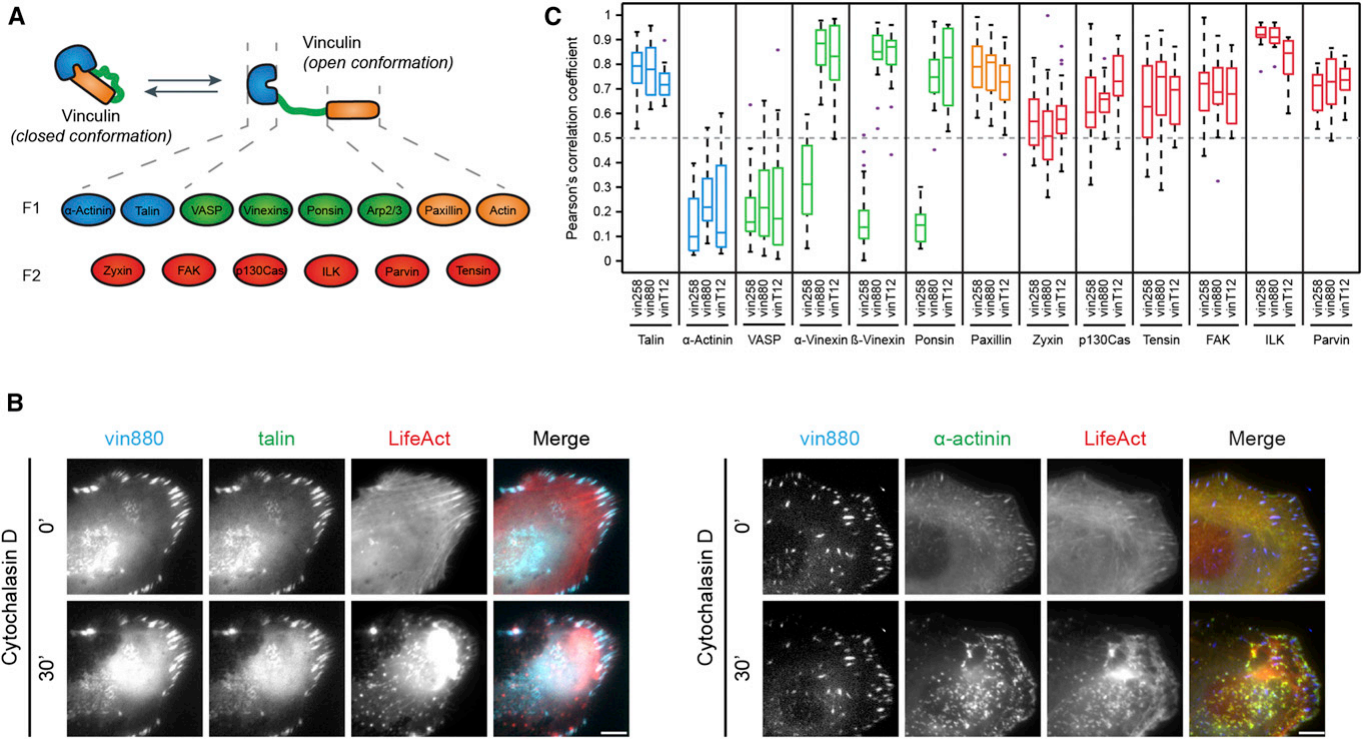
Vinculin Regulates the Recruitment and the Release of Core Proteins of the FA Network (A) Schematic of vinculin and its reported direct binding partners for the head (blue), neck (green), and tail (orange) regions (F1 generation). Indirectly associated FA core proteins that may bind through the F1 generation proteins are displayed as the F2 generation in red. (B) Images from live recordings of U2-OS cells expressing vin880-CFP, talin-YFP, and LifeAct-mRFP (left panel) or vin880-CFP, α-actinin-YFP, and LifeAct-mRFP (right panel). Note that vin880 stabilizes talin, but not α-actinin, in FAs when intracellular tension is released during cytochalasin D treatment (Movie S2). Scale bar represents 5 μm. (C) Pearson’s correlation analysis after actin disruption using Y-27632 in *MEFvin*^*−/−*^ cells was used to quantify the extent of colocalization of vinculin constructs with indicated FA proteins. Note the high correlations of all FA stabilizing vinculin forms with talin, paxillin, zyxin, p130Cas, ILK, parvin, FAK, and tensin and the low correlation with α-actinin and VASP. High correlations of α-vinexin, β-vinexin, and ponsin are dependent on the presence of the proline-rich neck region in vinculin.

The ability of active vinculin constructs to stabilize FAs in the absence of actomyosin tension allowed us to study whether other adhesion components are influenced by vinculin activity (Figure 2A). To explore which proteins are controlled by vinculin and which domains of vinculin are involved, we coexpressed each of the vinculin constructs tagged with one fluorophore together with another core FA protein tagged with a different fluorophore, before treating cells with Y-27632 or cytochalasin D. Two examples of such experiments are shown in Figure 2: First, talin, which provides the functional link to integrins and whose presence is a prerequisite for vinculin localization to FAs [8, 9], remained colocalized with vin880 after cytochalasin D treatment (Figure 2B and Movie S2). Pixel-by-pixel quantification of colocalization between vin880 and talin in *MEFvin*^*−/−*^ cells after drug treatment revealed a high Pearson’s correlation coefficient, indicating almost identical colocalization (Pearson's score ∼ 0.8) (Figure 2C). Second, and in contrast to talin, α-actinin, which binds the vinculin head, left FAs following cytochalasin D treatment and colocalized with the disrupted actin cytoskeleton (Figure 2B and Movie S2). The Pearson’s correlation coefficient for vin880 and α-actinin was correspondingly low (Pearson score < 0.3), an observation that challenges the proposed role of α-actinin in vinculin binding and activation [23].

Of the reported vinculin neck-binding proteins (Figure 2A), α-vinexin, β-vinexin, and ponsin remained in FAs after cytochalasin D treatment (Pearson's score ∼ 0.8) only when coexpressed with vinculin forms that contained the neck region, but not with vin258, which lacks this domain (Figure 2C). Thus, the presence of vinculin drives the recruitment of the vinexin family of proteins to FAs. In contrast, VASP disappeared from FAs under actin-disrupting conditions regardless of whether the cells expressed active vinculin mutants containing the neck region or not ([24] and Figure 2C). Arp2/3 [25] was not detected in FAs either before or after treatment with actin-destabilizing drugs.

Interestingly, paxillin had a high correlation score (Pearsons score ∼ 0.8) with vin258 and vin880 (Figure 2C) despite the fact that these constructs lack the reported binding site in the vinculin tail [26]. Performing these measurements in vinculin-deficient cells excluded the possibility of paxillin recruitment via dimerization of vin880 or vin258 with endogenous vinculin. This result suggests that active vinculin stabilizes paxillin in FAs in an indirect manner via other proteins within the adhesome network.

Among the FA proteins that have not been reported to bind vinculin directly, we found that focal adhesion kinase (FAK), ILK, parvin, p130Cas, zyxin, and tensin were stabilized by all active vinculin constructs following treatment of cells with Y-27632 (Figure 2C) or cytochalasin D (data not shown). Because they were also stabilized in FAs in cells expressing just the talin-binding vin258 D1 domain, we assume that, as for paxillin, the stabilization of these proteins in FAs is driven indirectly by vinculin via talin. To test whether the stabilization of FAs by active vinculin is observed in different cell lines, we repeated our coexpression experiments with essentially the same results in NIH 3T3 mouse fibroblasts and U2-OS human carcinoma cells (data not shown).

The exogenous expression of GFP-tagged proteins needs to be tightly controlled in order to avoid artifacts. To ensure that the exogenous expression of the different proteins mentioned above (e.g., FAK, ILK, etc.) did not contribute to FA stability, we expressed them in U2-OS human carcinoma cells without coexpressing active forms of vinculin and under tension-releasing drugs.

None of the expressed constructs were found to stabilize FAs by themselves (Figure S2A). Furthermore, we could confirm these results when staining for endogenous levels of vinculin-binding partners instead of coexpression (Figure S2B).

Collectively, these data indicate that vinculin activity governs the recruitment of the vinexin family of proteins to FAs and the release of a large number of other core FA proteins (Figure 2A). Because FAs in cells expressing vinFL disassemble under tension-releasing conditions, our experiments demonstrate that the inactivation of vinculin is required to release the majority of FA components tested from adhesion sites.

### Tension Is Required to Maintain Vinculin in FAs

**Figure 3:**
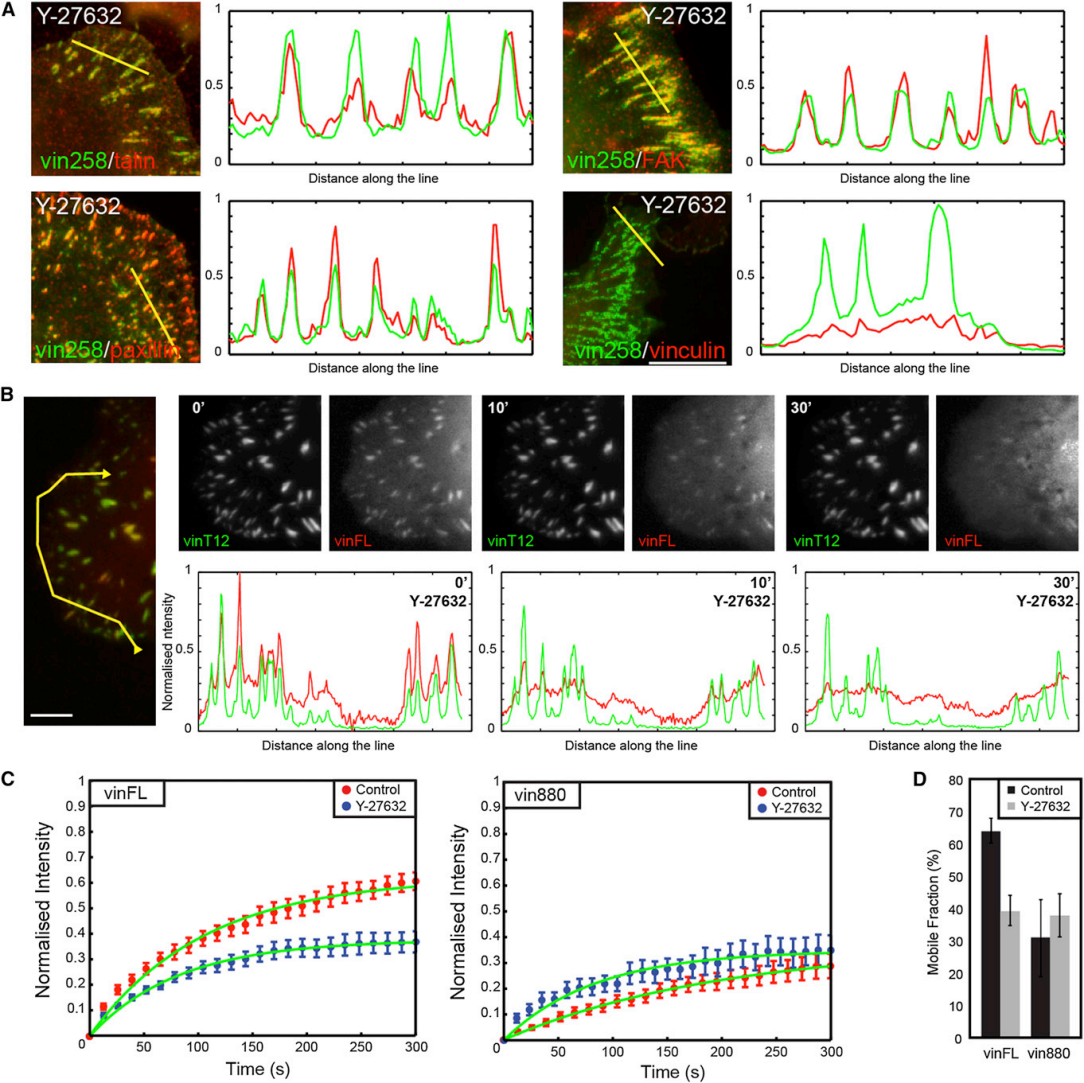
Intracellular Tension Is Required to Maintain Full-Length Vinculin in FAs (A) Expression of vin258 in U2-OS cells in the presence of Y-27632 prevents the release of endogenous talin, paxillin, and focal adhesion kinase (FAK), but not vinculin, in FAs. Note the overlap of the fluorescence intensity peaks for vin258 colocalization with talin, paxillin, and FAK and the absence of overlap of the profiles for GFP-vin258 with endogenous vinculin. Scale bar represents 5 μm. (B) Panels of time-lapse recordings of a U2-OS cell expressing vinFL-mCherry and vinT12-GFP during Y-27632 treatment (refer to Movie S3 for the full time course). Intensity profiles of vinT12 (green) and vinFL (red) along the yellow line at indicated time points are displayed below the images. During the drug treatment, the profile corresponding to vinT12 remains stable, and the matching peaks in the profile of vinFL decreases at the same time as the overall background increases. Intensities were corrected for photobleaching using a histogram-matching method and normalized using the total fluorescence in each channel. Scale bar represents 5 μm. (C and D) FRAP experiments were performed in *MEFvin*^*−/−*^ cells expressing either vinFL-GFP or vin880-GFP and treated with Y-27632. Note the slower fluorescence recovery in FAs of cells expressing vinFL when treated with Y-27632. (E) Quantification of the mobile fraction of indicated GFP-fusion proteins in FAs. Note the significant decrease in mobile fraction of vinFL-GFP after treatment with Y-27632. No statistically significant difference was observed in the mobile fraction of vin880 upon drug treatment (±SEM; p < 0.01).

The experiments presented in Figure 1 show that active vinculin bypasses the requirement for tension to maintain stable FAs. Others have shown that vinculin itself is involved in the transmission of actomyosin-mediated tension [15]. The question therefore arises whether actomyosin-mediated tension is directly involved in the activation of vinculin and its localization to FAs. To test this, we expressed FA-stabilizing vinT12 or vin258 in U2-OS cells containing endogenous vinculin. Following treatment of cells with the ROCK inhibitor Y-27632, the stabilized FAs remained positive for talin, paxillin, and FAK (Figures 3A and S2B). However, they lacked endogenous vinculin, which was found diffusely distributed in the cytoplasm (Figure 3A). To confirm these results, we coexpressed either the mCherry-vinT12/GFP-vin880 pair or mCherrry-vinT12/GFP-vinFL. Whereas both vinT12 and vin880 were present in FAs in equal amounts before and after treatment of cells with Y-27632 (Figure S3), essentially all vinFL was lost from FAs after 20 min following ROCK inhibition (Figure 3B and Movie S3). The diffuse localization throughout the cytoplasm suggested that vinFL, upon tension release, converts from an active conformation localizing in FAs back into a cytoplasmic inactive conformation which cannot be incorporated into FAs [17]. To further test this, we performed FRAP experiments in *MEFvin*^*−/−*^ cells and showed that the recruitment, and therefore the mobile fraction of vinFL, significantly decreased 5 min after Y-27632 treatment. No significant changes in mobility were observed for vin880 (Figure 3C). Taken together, these results establish that actomyosin-mediated forces directly acting on vinculin are required to maintain vinculin in an activated FA-stabilizing state.

### Vinculin Activity Stabilizes the Talin-Integrin Complex in an Active Conformation Leading to Increased Cell Adhesion

We have previously shown that vinculin decreases the mobility of talin and integrins in FAs [11, 20] and hypothesized that active vinculin holds integrins in an extended, ligand-bound conformation. To test the impact of vinculin on integrin activation and the potential functional consequences for cell adhesion, we performed force spectroscopy measurements. In these assays, we attached cells to a cantilever tip coated with a polyphenolic protein solution. The attached cell was then pressed for 5 s onto a fibronectin-coated glass-bottom dish, and adhesion strength was determined by measuring the retraction forces needed to detach the cell from the ligand (Figure S4A). The short time frame of attachment does not allow the cell to organize its actomyosin machinery, and therefore intracellular tension produced by myosin contraction was absent in these cells.

**Figure 4:**
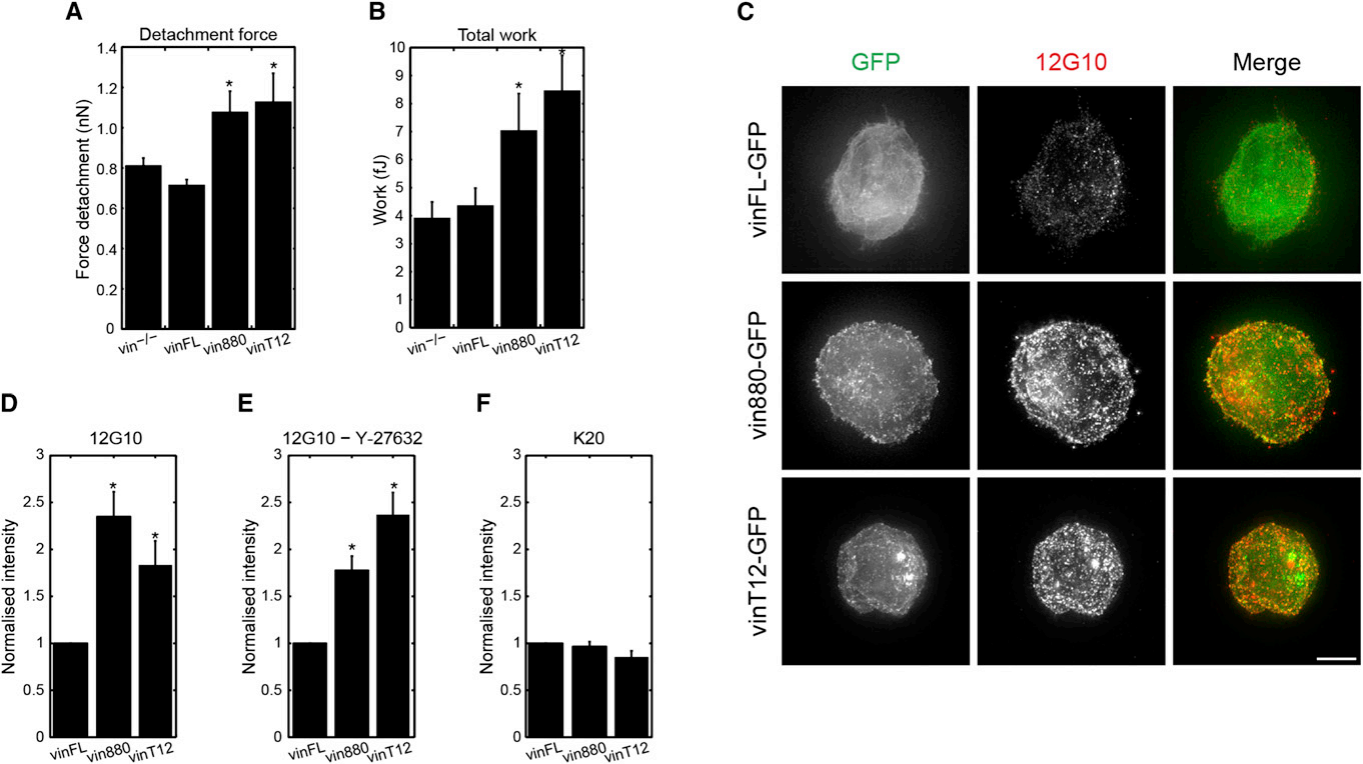
Active Vinculin Holds Integrin in an Active Conformation (A and B) Expression of vinT12 or vin880, but not vinFL, in *MEFvin*^*−/−*^ cells increases the maximal detachment force and working energy when compared to control cells. (A schematic of the experimental setup is in Figure S4A.) (C) U2-OS cells were seeded on poly-D-lysine to hold them in a nonspread and tensionless shape. Note the increase in antibody staining for activated β1-integrins (12G10) upon expression of vin258, vin880, and vinT12 compared to control cells expressing vinFL (Movie S4). Scale bar represents 5 μm. (D and E) Quantification of the intensity of 12G10 labeling under control conditions (D) and after addition of Y-27632 (E). (F) Quantification of total β1-integrin labeling under indicated conditions, using an antibody that binds to all conformations of β1-integrins (K20). All quantifications are expressed as ratios over the vinFL sample in the same condition (p < 0.01; ±SEM, n ≥ 15 cells from two independent experiments per condition).

Expression of vin880 or vinT12 in *MEFvin*^*−/−*^ cells resulted in an increase in the maximal force and the total work energy required to detach from FN compared to untransfected cells (Figures 4A and 4B). No increase was observed in cells reexpressing wild-type vinculin, thus demonstrating that expression of active vinculin stabilizes integrins in a high-affinity, ligand-binding state (Figures 4A and 4B). No adhesion was detected on BSA, indicating that the result was an integrin-mediated effect (Figure S4).

We confirmed these results with an imaging approach using U2-OS cells and integrin activation-status-specific anti-integrin antibodies [27]. To mimic cell attachment in the same tensionless and round morphology as on the cantilever, we plated cells for 7 min on poly-D-lysine, a substrate that does not engage integrins and does not support cell spreading. Staining with anti-integrin β1 antibody 12G10, which detects active integrins in an open conformation, revealed abundant clusters of active integrins on cells expressing all active forms of vinculin compared to cells expressing vinFL (Figure 4C and Movie S4). Quantitative measurements show that the high-intensity 12G10-positive clusters colocalized with the expressed active vinculin forms (Figure 4D). To rule out the possibility that force contributed to integrin activation, we assessed 12G10 binding in the presence of Y-27632 (Figure 4E). Under these conditions, cells expressing all active forms of vinculin showed increased 12G10 binding compared to vinFL, indicating that active vinculin holds integrins in an active conformation, in the absence of tension. Staining with K20, an antibody that detects all forms of β1 integrin, confirmed that the results with 12G10 were due to conformational changes in integrin and not due to increased integrin expression (Figure 4F).

Taken together, these experiments clearly demonstrate that vinculin activation influences integrin activity and cell adhesion. Moreover, the expression of vinFL in *MEFvin*^*−/−*^ cells did not increase integrin-mediated adhesion in round cells (Figures 4A and 4B): the cells did not display anchorage points to the ECM, had no actin stress fibers, and therefore lacked intracellular tension. Overall, these data confirm that actin-mediated tension is required for vinculin activation and translocation to FAs.

### The Controlled Release of FA Proteins through the Regulation of Vinculin Is Important for Polarized Cell Migration

**Figure 5:**
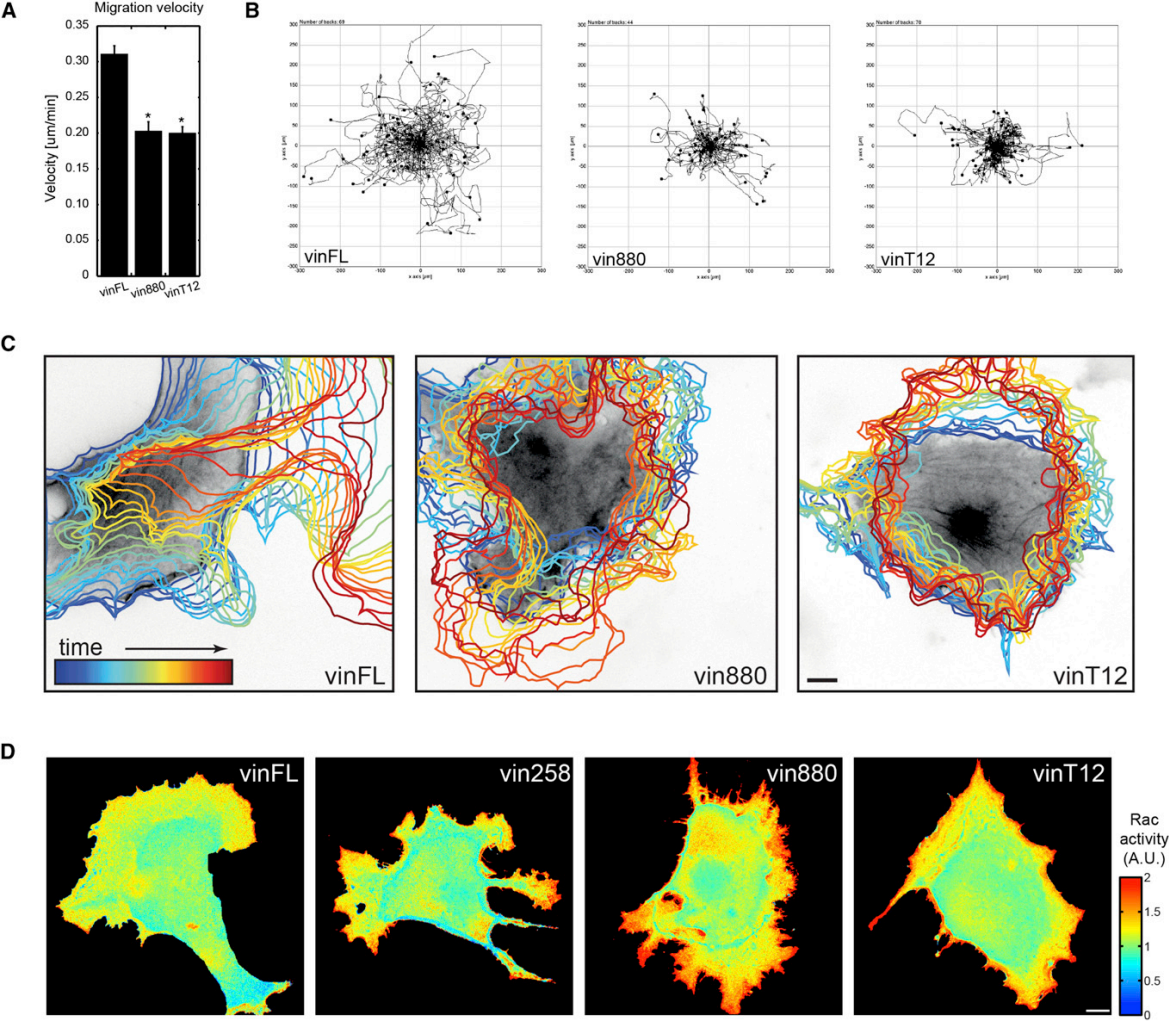
Expression of FA-Stabilizing Vinculin Impairs Polarized Cell Migration (A) Cell velocities of B16F10 cells expressing indicated constructs when plated on laminin. (B) The plotted trajectories of 24 hr time-lapse experiments outline the decreased migration rate of cells expressing vin880 and vinT12 when compared to vinFL-expressing cells (experiment representative of three replicates; n = 69, 44, and 70 for vinFL, vin880, and vinT12 respectively; ±SEM; p < 0.01). (C) High-magnification recordings of cells expressing indicated constructs were taken to outline potential differences in B16F10 polarity during migration. The color-coded shape outlines indicate representative protrusive activities recorded over a period of 2 hr (5 min intervals). Note the highly directional protrusion of vinFL-expressing cells leading to coordinated cell migration and the random unpolarized cell edge protrusions in cells expressing vin880 and T12 (Movie S5) (example representative of n > 10 cells from three independent experiments). Scale bar represents 5 μm. (D) FRET-based Rac activation sensors (Raichu-Rac) coexpressed with mCherry-tagged vinculin constructs outline localized Rac1 activity in B16F10 plated on laminin. In cells coexpressing vinFL, Rac1 activity is visualized by a color shift toward red and usually found at a single protrusive edge at the front of a migrating cell. In cells expressing active vinculin constructs (vin258, vin880, and vinT12), all the numerous peripheral protrusions showed sites of intense Rac activity (examples representative of n > 10 cells from two independent experiments). Scale bar represents 5 μm.

Because FA proteins are subject to continuous turnover during physiological processes such as cell migration [2–4], the above data imply that the inactivation of vinculin will be required to achieve efficient cell migration. To determine whether the FA-stabilizing effect of active vinculin affects cell migration, we tracked the movement of B16F10 melanoma cells. These cells were used because they migrate on laminin in a fast and highly polarized fashion [28, 29]. As expected, after the analysis of the cell adhesion strength described above, we observed a significant decrease in migration speed of B16F10 cells expressing vin880 or vinT12 constructs compared to those expressing vinFL (Figures 5A and 5B). Interestingly, we found dramatic differences in cell polarity associated with the decrease in migration speed. Whereas B16F10 cells expressing vinFL migrated in a polarized fashion, cells expressing the FAs stabilizing vinculin forms showed random unpolarized ruffling in all directions. *MEFvin*^*−/−*^ cells expressing the different vinculin forms plated on laminin showed a similar polarization phenotype to the B16F10 cells (Movie S5). However, *MEFvin*^*−/−*^ cells without vinculin behaved differently, in that they exhibited an unpolarized mode of migration with a high turnover of adhesion sites associated with filopodial structures, as was reported previously [30] (Movie S6).

We speculated that the unpolarized protrusions in cells expressing the FA stabilizing forms of vinculin were due to mislocalized Rac1 activity, possibly triggered through the trapping of proteins such as paxillin that regulate this pathway [31]. Indeed, using a Rac1 FRET-based activity reporter [32], we observed polarized Rac1 activity in the leading edge of a migrating cell upon coexpression with vinFL and unpolarized Rac1 activity localized circumferentially in cells expressing mCherry-tagged vin258, vin880, or vinT12 (Figure 5D). The importance of Rac1 for this protrusive activity was confirmed by the coexpression of vinculin constructs with dominant negative Rac1 (Rac1N17) [33], which blocked all protrusive activities in cells expressing the active vinT12 construct (Figure S5). Overall, these data demonstrate that the coordinated spatiotemporal activation and inactivation of vinculin is essential for Rac1-mediated polarized cell migration.

### Vinculin Coordinates Stretch-Induced Cell Polarization via Its Link to Actin

Complex molecular feedback loops direct polarized reorganization of cells, and the results above demonstrate that constitutive activation of vinculin perturbs directional cell motility. Interestingly, in these assays all active forms of vinculin perturbed cell polarity, irrespective of the presence of the vinculin neck and tail regions. Previous publications demonstrated that vinculin is exposed to forces and that the actin-binding tail region is of particular importance for force transmission [15]. To test the functional relevance of vinculin and its potential to transmit force, we applied cyclic stretching forces to cells, which in normal conditions leads to the reorientation of FAs with their long axis perpendicular to the direction of the stretching force [34].

**Figure 6:**
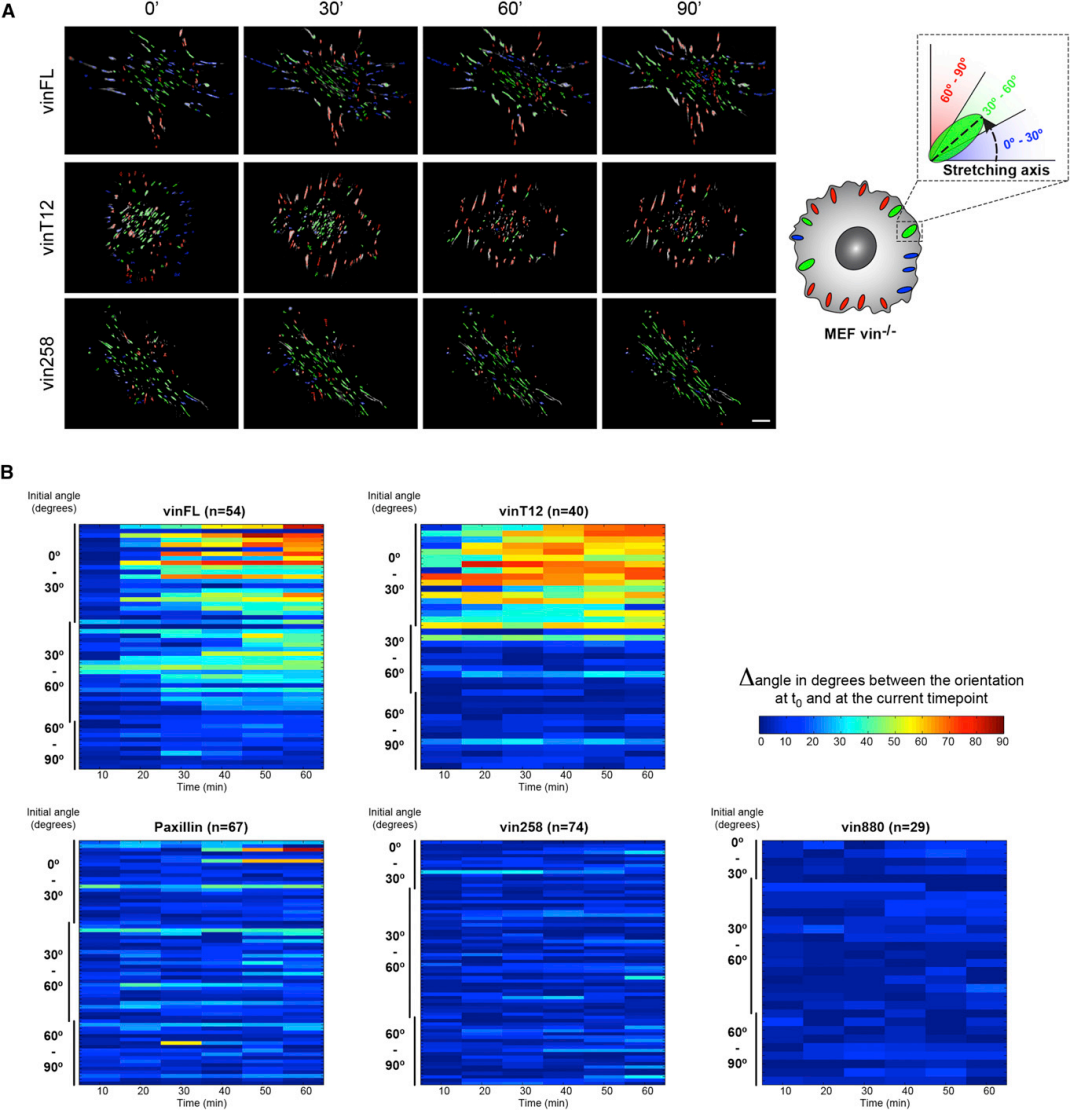
The Actin-Binding Site Present in Vinculin Tail Is Important for Stretch-Induced Reorganization of Cell Polarity (A) Still images from time-lapse recordings of *MEFvin*^*−/−*^ cells undergoing cyclic stretching, expressing indicated vinculin constructs (see Movie S7 for higher resolution). The FAs are color coded according to the angle between their main axis and the stretching axis: red for FAs in an angle between 0° and 30° to the stretching axis, green between 30° and 60°, and blue between 60° and 90°. Note the reorientation of FAs in cells expressing vinFL and vinT12 and the static behavior of FAs expressing vin258. FAs of cells expressing vinT12 reorganized faster than in vinFL-expressing cells, having reached their final position within 30 min of stretching (Movie S7). Scale bar represents 5 μm. (B) Heat maps display the change in reorientation of the FAs (Δangle) relative to their initial angle (y axis) and during the time course of the stretching (x axis). The amplitude of the reorganization is color coded (see rainbow color bar). Most of the FAs with an initial angle between 0° and 60° to the stretching axis readily reoriented during the time of cyclic stretching. In contrast, FAs of cells without vinculin (*MEFvin*^*−/−*^ cells expressing paxillin-GFP as adhesion marker) or cells expressing vinculin constructs lacking the actin-binding site (vin258, vin880) were impaired in FA reorientation. The faster reorganization of FAs in cells expressing vinT12 highlights the importance of the vinculin-actin link in the transmission of stretch-induced forces.

As expected, *MEFvin*^*−/−*^ cells expressing vinFL reoriented their adhesion sites perpendicular to the stretching axis to a level similar (close to 90° from the stretching axis) to that previously reported for NIH 3T3 fibroblasts [34] (Figure 6A and Movie S7). The heat maps in Figure 6B indicate that only those FAs with an initial angle between 0° and 30° to the stretching direction reoriented during the period of stretching. FAs between 30° and 60° were less responsive, and those that were almost perpendicular (60° to 90°) to stretching forces remained unchanged. FAs in *MEFvin*^*−/−*^ cells responded weakly, and only a very small subset of their adhesions reoriented perpendicular to the stretching axis, irrespective of their initial angle to the direction of stretch. Similarly, most FAs in cells expressing either vin258 or vin880 (both of which lack the actin-binding vinculin tail) failed to reorient during the stretching process (Figure 6). In contrast, FAs in cells expressing the constitutively active vinT12 [35], where the actin-binding site in the tail is exposed, repolarized with faster kinetics than cells expressing wild-type vinFL (Figure 6B). In these cells, FAs with an initial angle of 0° and 30° had reoriented after 30 min, whereas the majority of those in cells expressing vinFL required 60 min for maximal reorientation. Thus, unlike the perturbations of polarized cell migration induced by all active forms of vinculin, the response to stretching forces leading to cellular reorganization of the adhesion machinery was controlled by the presence of the actin-binding tail in vinculin. Without the actin-binding tail, force transduction to the actin cytoskeleton remained inefficient. Overall, these data indicate that vinculin plays a major role in the transmission of stretching forces and that the link between the vinculin tail and the actin cytoskeleton is essential in the transmission of stretch-induced force signals leading to cell reorientation.

## Discussion

Vinculin is essential for embryonic development and efficient cell adhesion and migration [30], but the molecular mechanisms that control these processes are not well understood. In this study, we provide new insights into how vinculin regulates cell adhesion, migration, and cell polarization. We found that (1) actomyosin-mediated tension is required for vinculin activation and localization to FAs, (2) vinculin regulates the recruitment and release of core FA proteins in a force-dependent manner, (3) vinculin controls the activity of integrins via its interaction with talin, (4) vinculin thereby regulates polarized cell migration, and (5) vinculin transmits force-induced signals between integrin-ECM adhesions and the cytoskeleton that are required for force-induced cell repolarization.

Current models for vinculin activation include PIP_2_ or actin as coactivators with talin [12]. Our experimental data support a model whereby actomyosin-mediated forces are essential for vinculin activation and localization to mature FAs. This is based on the observations that endogenous vinculin, in contrast to most of the other core FA proteins studied here, leaves FAs when tension is released even when FAs are stabilized through the expression of a constitutively active form of vinculin. This feature suggests that vinculin-mediated signaling pathways remain active only in areas where tension is maintained. During cell migration, vinculin activation occurs at the protruding cell front where focal complexes mature into tension-dependent FAs. In areas where tension is insufficient to keep vinculin in an extended active conformation, FAs become labile and disassemble. Such a scenario would explain the transient formation of adhesions in migrating cells, whereby adhesion sites grow in a restricted area at the cell front with highest Rho activity and tension. FAs would then disassemble in the lamella in front of the nucleus where Rho activity, and subsequently tensile forces, are reduced [36].

The stabilization of adhesions by constitutively active vinculin is mediated by its effect on the talin-integrin complex. Evidence for this is threefold: (1) the presence of talin is essential for vinculin localization in FAs [8, 9], (2) the A50I mutation in vinculin that reduces talin binding inhibits the FA stabilizing effect of vinculin, and (3) the minimal talin-binding domain of vinculin (vin258) is sufficient for FA stabilization, which is compromised by an A50I mutation [20].

The costabilization of many other proteins within FAs by active vinculin reveals that vinculin coordinates the recruitment of the vinexin family of proteins and, more importantly, the release of FA components that belong to the basal layer of the FA structure. Because many of the proteins costabilized in FAs, such as FAK, paxillin, zyxin, ILK, and p130Cas, have no binding sites on vin258, it is clear that they are recruited through indirect associations driven by the action of vinculin on the talin-integrin complex.

We further show that the recruitment of α- and β-vinexin and ponsin occurs through direct interaction with the neck region of vinculin, thereby confirming previous observations [17, 37, 38]. Other reported neck-binding proteins, such as Arp2/3 and VASP [24, 25], failed to correlate well with active vinculin following tension release. This may not be surprising for Arp2/3, given that it is only present transiently in nascent adhesions and absent from FAs where fully activated vinculin is found [25]. The absence of VASP could be explained by a relocalization of VASP to sites of actin polymerization following the treatment with Y-27632. Indeed VASP, under ROCK inhibition, strongly localizes to actin at protruding sites of the cell.

The absence of α-actinin from vinculin-stabilized FAs was somewhat surprising, given that it reportedly binds vinculin and has been suggested to play a role in vinculin activation [23]. Our observations that α-actinin localized to the actin cytoskeleton fraction are consistent with recent studies using superresolution microscopy, where α-actinin was found associated with actin filaments and not in the FA plaque [39].

FAs are sites of force transduction, and the role of vinculin in FA repolarization during cyclic stretching of the substrate differs from its role during cell migration. The migratory behavior of cells is dependent on the assembly and disassembly of adhesion receptor complexes and the establishment of polarized protrusive activity—both of which are regulated by vinculin. Atomic force spectroscopy measurements and tension inhibition experiments show that vinculin influences integrin activity via talin. Our cell motility data demonstrate that constitutive activation of vinculin leads to unpolarized protrusive activity regulated by mislocalized Rac1 activity. Overall, these data suggest that cycles of vinculin activation and inactivation control the recruitment and release of FA proteins, which are required for polarized cell migration.

Our data using cyclic stretching techniques define vinculin as a force-transducing protein required for the efficient reorientation of adhesions under force, leading to the repolarization of cells.

Vinculin directly experiences force in live cells [15], and we show here that its association with F-actin is essential for FAs to respond to mechanical forces. This finding is surprising, given that FAs contain other actin-binding proteins, such as talin [40, 41], the ILK-PINCH-parvin ternary complex [42], zyxin [43], and α-actinin [44], which provide additional integrin-actin connections. Because cells lacking vinculin still assemble FAs [30], these proteins may be sufficient to regulate the basic cell adhesion machinery. However, the additional stability of FAs mediated by vinculin may provide a much increased cohesion that is essential to maintain the integrity and function of tissues that encounter increased mechanical force, for example, under stretching conditions. In cadherin-mediated cell-cell adhesions, vinculin may have a role similar to its role in FAs, where it is recruited in a force-dependent manner to increase stability [45]. Overall, these observations may explain the compromised heart function in mice when cardiomyocytes are deficient in vinculin [46].

In conclusion, we show that vinculin controls the transmission of force from the cell’s extracellular environment through the talin-integrin complex to the actomyosin machinery. We propose a model whereby, in migrating cells, vinculin coordinates the recruitment and release of core FA proteins in time and space. The main trigger for vinculin activation is actomyosin-mediated force, which drives the stabilization of core components within FAs. In subcellular areas of high force, vinculin stabilizes signaling components (e.g., paxillin), resulting in local activation of integrin-mediated cell adhesion and Rac1-mediated cell protrusions. In subcellular areas of low tension, vinculin cannot maintain an active conformation and is therefore released. As a consequence, cell-matrix adhesions become labile and disassemble. This model is in good agreement with data showing that the appearance of FAs in migrating cells is restricted to an area close to the leading edge, which correlates with regions of high tensile forces generated by the interaction with the extracellular environment [47, 48].

Depending on the tissue type, cells may be exposed to continuous forces from their external environment. In such a scenario, involving “outside-in” signaling, a high proportion of vinculin might be continuously activated, and its role could switch from regulating FA assembly and disassembly to providing a stable force-transducing connection between the integrin-talin complex and the actin cytoskeleton. Under these conditions, the direct binding of vinculin to talin and actin enables an efficient transfer of mechanical cues from the outer environment to the inside of the cell, thus permitting cells to react to changing mechanical stimuli.

## Experimental Procedures

### Cells, Media, and Reagents

B16F10 mouse melanoma cells, vinculin-deficient mouse embryonic fibroblast (*MEFvin*^*−/−*^) cells, and U2-OS osteosarcoma cells were cultured at 37°C in Dulbecco’s modified Eagle’s medium (Sigma-Aldrich) supplemented with 10% fetal bovine serum (Lonza Cologne GmbH), 2 mM L-glutamine, and nonessential amino acids (all from Sigma-Aldrich). *MEFvin*^*−/−*^ cells were provided by David Critchley (University of Leicester, Leicester, UK [30]); the other cell lines were purchased from LGC-ATCC. Unless stated otherwise, the live-cell imaging experiments were performed in carbonate-free Ham’s F-12 media supplemented with L-glutamine, 25 mM HEPES, and penicillin-streptomycin (all from Sigma-Aldrich). When applied, Y-27632 was used at 100 μM diluted in water and cytochalasin D at 2 μM, and blebbistatin was used at 50 μM diluted in DMSO.

See Supplemental Experimental Procedures for reagents and plasmids used, imaging procedures, stretching experiment techniques, data analyses, and other experimental details.

## Supplemental Information

Document S1. Figures S1–S5 and Supplemental Experimental Procedures

Movie S1. Related to Figure 1. Recordings of FAs from *MEFvin*^−*/*−^ Cells Expressing Indicated Constructs when Treated with the Rho-Kinase Inhibitor Y-27632 or the Actin-Disrupting Agent Cytochalasin D The top panel shows treatment with Y-27632, and the bottom panel shows treatment with cytochalasin D. Note that only vin258, vin880, and vinT12 stabilize FAs under the tension-releasing conditions. An A50I mutation that reduces the vinculin-talin interaction strength abolishes the FA stabilizing effect of these constructs. Video displays images taken every minute. Images are played back at 5 frames/s.

Movie S2. Related to Figure 2. Time-Lapse Recording of a U2-OS Cell Coexpressing Talin-YFP, Vin880-CFP, and LifeAct-mRFP or α-Actinin-YFP, Vin880-CFP, and LifeAct-mRFP and Treated with Cytochalasin D Note that both vin880 and talin remain stable, identically localizing over the course of stress fiber disassembly. In contrast, α-actinin disappears from FAs and follows the actin positive structures in the cell. Video displays images taken every minute. Images are played back at 10 frames/s.

Movie S3. Related to Figure 3. Time-Lapse Recording of U2-OS Cell Coexpressing VinFL and VinT12 Cells are treated with 100 μM of Y-27632 at the beginning of the movie. Time is indicated at the top left in minutes. The red and green profiles reveal the normalized intensities of vinFL-mCherry and vinT12-GFP, respectively, along the yellow line drawn on the merged image. Images are played back at 10 frames/s.

Movie S4. Related to Figure 4. Three-Dimensional Reconstruction of U2-OS Cells Expressing the Indicated Vinculin Constructs Cells were costained with 12G10 antibody to detect activated β1-integrins. Only cells expressing the FA stabilizing vinculin forms vin880 or vinT12 were able to maintain clusters positive for active β1-integrins in round cells in the absence of intracellular tension. All immunofluorescence images were captured with the same exposure times for direct comparison and quantification of immunostainings.

Movie S5. Related to Figure 5. Recordings of FAs and Actin Dynamics from B16F10 Cells Coexpressing Indicated Vinculin Construct and LifeAct-mRFP when Plated on Laminin Cells expressing active vinculin constructs (vin880 or vinT12) exhibit protrusive activity all around the cell, whereas the cell expressing vinFL displays space- and time-restricted protrusive activity. The protrusive activity is highlighted by the color-coded cell shape outlines during cell recordings in time. Video displays images taken every minute. Images are played back at 10 frames/s.

Movie S6. Related to Figure 5. Cells Change Their Migration Mode after Reexpression of Vinculin *MEFvin*^−*/*−^ cells expressing the indicated constructs are plated on 5 μg/ml of laminin and imaged using TIRF microscopy (or epifluorescence microscopy for LifeAct-mRFP only). Reexpression of full-length vinculin induces the rescue of polarized migratory behavior, which is strikingly different from *MEFvin*^−*/*−^ cells and characterized by an increase in size and number of FAs. The expression of any active vinculin construct (vin880 or vinT12) leads to a more static phenotype, where cells protrude in every direction, but fails to migrate effectively. This change of phenotype is comparable to what was characterized for B16F10 cells (see Figure 5C). Images are played back at 10 frames/s.

Movie S7. Related to Figure 6. Time-Lapse Recording of *MEFvin*^−*/*−^ Cells Expressing the Indicated Vinculin Construct during Substrate Stretching Cells are expressing the GFP-tagged vinculin construct as indicated. The perimeter of each FA is a color-coded representation of the FAs based on their orientation at different time points during cyclic stretching. The orientation angle is determined as the angle between the main axis of each FA and the stretching axis (indicated with the yellow double arrow). Briefly, cells expressing vinT12 reorganize their FAs to become perpendicular to the stretching axis faster than cells expressing vinFL. FAs in cells expressing vin258 that lacks the actin-binding site in the vinculin tail fail to reorient during the stretching time course. The bottom right panel depicts typical examples of FA dynamics during the time course of the experiment. The green arrows point toward FAs whose orientation changes immediately under tension; the arrowhead points to a FA, which is first formed parallel to stretching forces but then switches orientation after an initial phase of protrusion. The red arrow points toward a typical FA in a cell expressing vin258, which does not change its reorientation after application of stretch. Images are played back at 2 frames/s.
