# Supplementary material for: Vinculin Regulates the Recruitment and Release of Core Focal Adhesion Proteins in a Force-Dependent Manner
Source: Curr Biol. 2013 Feb 18;23(4):271–81. doi: 10.1016/j.cub.2013.01.009 (PMC3580286; doi:10.1016/j.cub.2013.01.009)
Supplement: Document S1. Figures S1–S5 and Supplemental Experimental Procedures [file mmc1.pdf]

**Current Biology, Volume *23***

**Supplemental Information**

# Vinculin Regulates the Recruitment and Release of Core Focal Adhesion Proteins in a Force-Dependent Manner

**Alex Carisey, Ricky Tsang, Alexandra M. Greiner, Nadja Nijenhuis, Nikki Heath, Alicja Nazgiewicz, Ralf Kemkemer, Brian Derby, Joachim Spatz, and Christoph Ballestrem**

# Figure S1

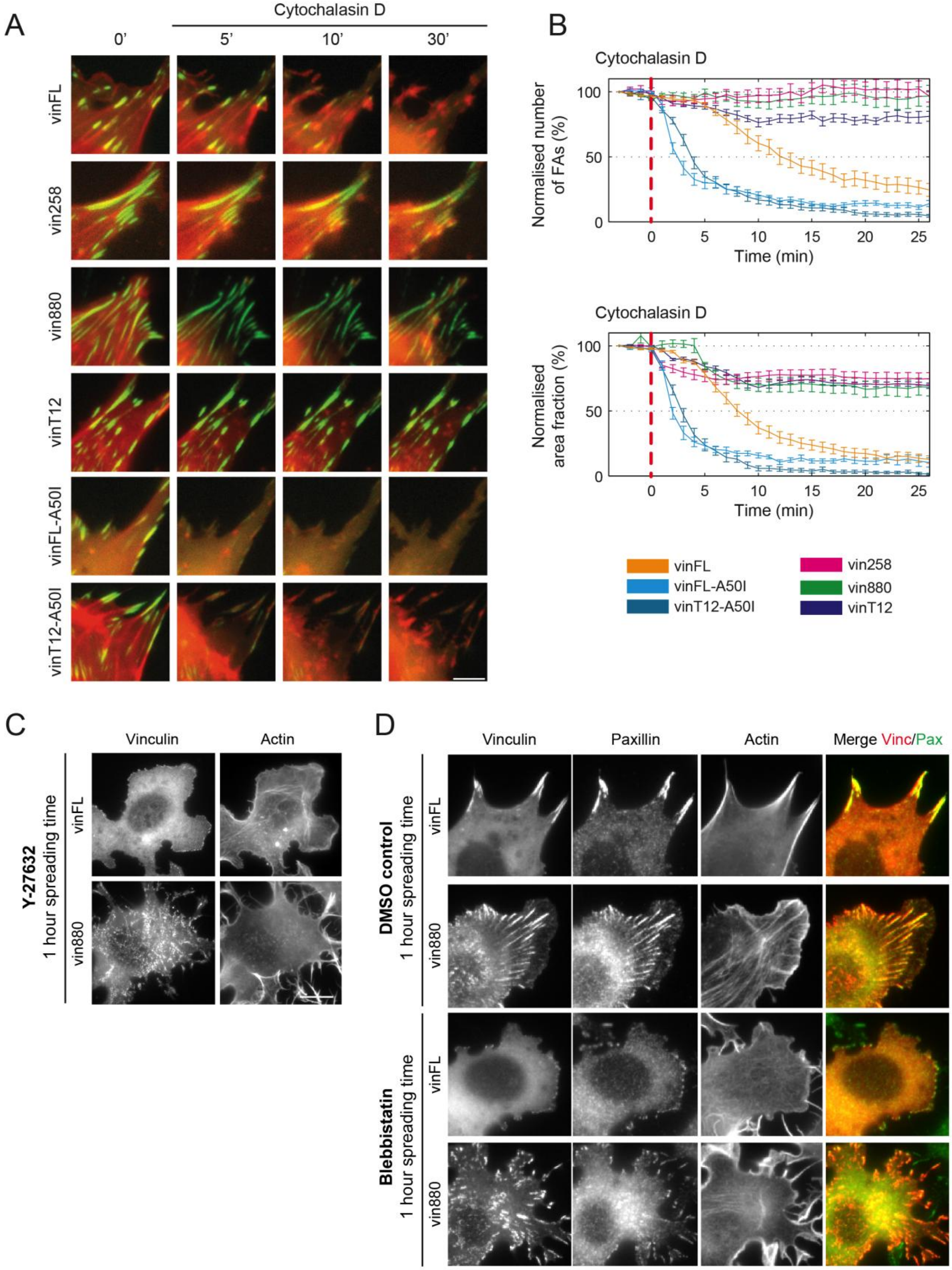
A
Cytochalasin D
0'
5'
10'
30'
vinFL
vin258
vin880
vinT12
vinFL-A50I
vinT12-A50I
B
Cytochalasin D
Normalised number of FAs (%)
Time (min)
Cytochalasin D
Normalised area fraction (%)
Time (min)
vinFL
vinFL-A50I
vinT12-A50I
vin258
vin880
vinT12
C
Vinculin
Actin
Y-27632
1 hour spreading time
vinFL
vin880
D
Vinculin
Paxillin
Actin
Merge Vinc/Pax
DMSO control
1 hour spreading time
vinFL
vin880
Blebbistatin
1 hour spreading time
vinFL
vin880

**Figure S1, Related to Figure 1. The Expression of Active Forms of Vinculin Stabilises FAs and Thereby Bypasses the Requirement of Intracellular Tension for FA Maintenance**

(A) Time-lapse images of vinculin-deficient MEFs expressing indicated vinculin constructs together with LifeAct-mRFP. Note that cytochalasin D treatment leads to the loss of FAs in cells expressing vinFL, vinFL-A50I and vinT12-A50I; in contrast, FAs remain stable in presence of vin258, vin880 and vinT12 (figure 1B-C for Y-27632 treatment and movie S1). Scale bar, 5 μm.

(B) Quantification of the normalised FA number and area fraction over the time-course of the drug treatment. The vertical red-dashed line indicates the beginning of the treatment. Error bars indicate ±S.E.M., n>10 cells from 2 independent experiments.

(C) Representative images of MEFvin$^{-/-}$ cells expressing vinFL-GFP or vin880-GFP treated with Y-27632, 30 mins before and one hour during spreading. Note the presence of vin880 positive FAs and the loss of vinFL during Y-27632 treatment. Scale bar, 5 μm.

(D) Representative images of MEFvin$^{-/-}$ cells expressing vinFL-GFP or vin880-GFP treated with DMSO control or blebbistatin, 30 mins before and one hour during spreading. Note the presence of vin880 positive FAs that colocalise with paxillin, and the loss of vinFL during blebbistatin treatment. Scale bar, 5 μm.

# Figure S2

A

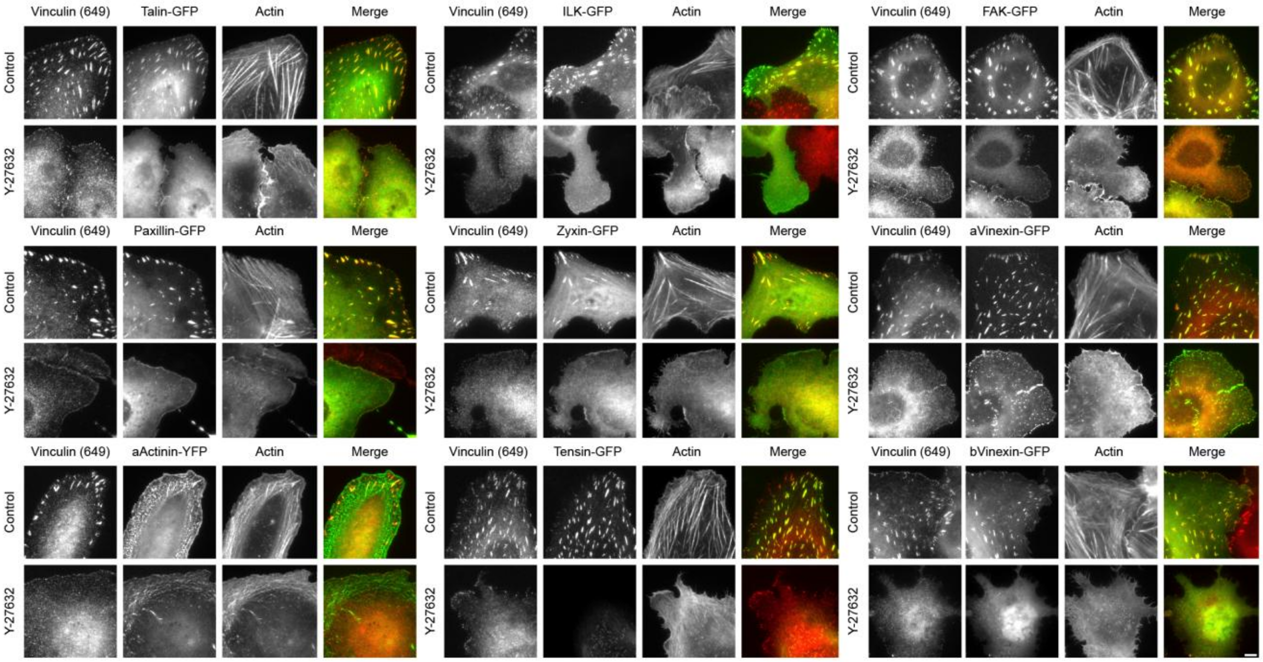
Vinculin (649)
Talin-GFP
Actin
Merge
Vinculin (649)
ILK-GFP
Actin
Merge
Vinculin (649)
FAK-GFP
Actin
Merge
Control
Y-27632
Vinculin (649)
Paxillin-GFP
Actin
Merge
Vinculin (649)
Zyxin-GFP
Actin
Merge
Vinculin (649)
aVinexin-GFP
Actin
Merge
Vinculin (649)
aActinin-YFP
Actin
Merge
Vinculin (649)
Tensin-GFP
Actin
Merge
Vinculin (649)
bVinexin-GFP
Actin
Merge

B

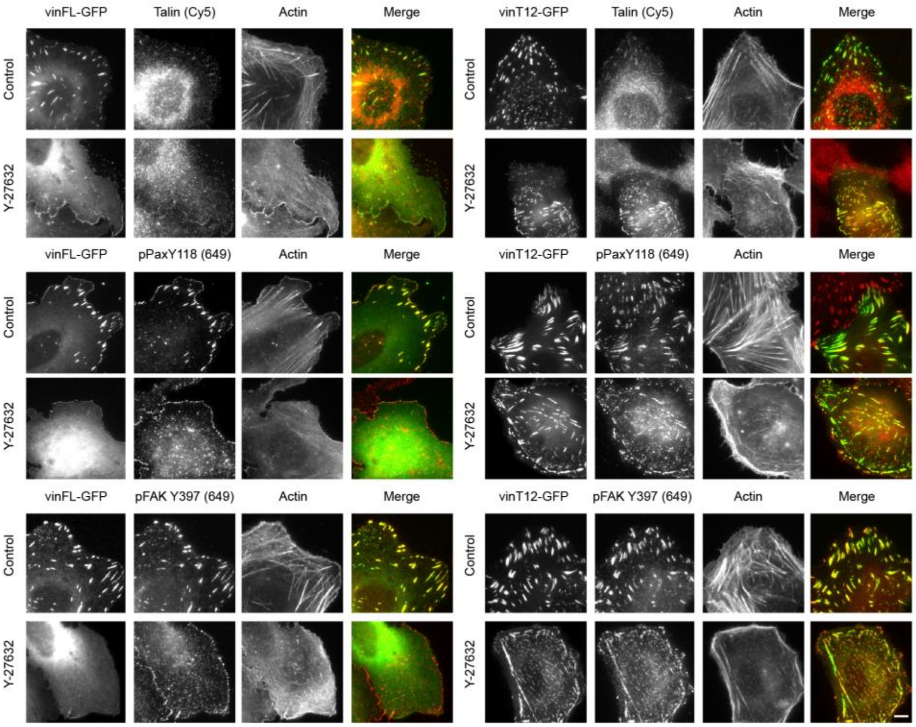
vinFL-GFP
Talin (Cy5)
Actin
Merge
vinT12-GFP
Talin (Cy5)
Actin
Merge
Control
Y-27632
vinFL-GFP
pPaxY118 (649)
Actin
Merge
vinT12-GFP
pPaxY118 (649)
Actin
Merge
vinFL-GFP
pFAK Y397 (649)
Actin
Merge
vinT12-GFP
pFAK Y397 (649)
Actin
Merge

**Figure S2, Related to Figure 2. The Exogenous Expression of GFP-Tagged FA Constructs Does Not Alter FA Behaviour under Tension-Releasing Conditions**

(A) In order to ensure that the overexpression of GFP-tagged proteins does not cause FA artefacts, we tested whether their expression in cells behave in a similar way as we would expect from endogenous proteins. To examine GFP-fusion constructs we expressed them in U2-OS cells stained for endogenous vinculin and actin. Staining before treatment with drugs show that all indicated proteins readily localise to FAs that are also positive for vinculin. After treatment with Y-27632 for 1 hour, all FAs disappeared and none of the overexpressed components stabilised FAs.

(B) Also the overexpression of vinculin full-length did not stabilize FAs. Under control conditions GFP-vinculin co-localises with endogenous talin, paxillin and FAK that are all released from FAs upon treatment with Y-27632. The expression of constitutively active full-length vinculin (vinT12) stabilised FAs and endogenous talin, FAK and paxillin therein despite the release of intracellular tension upon Y-27632 treatment. Scale bar, 5 μm.

# Figure S3

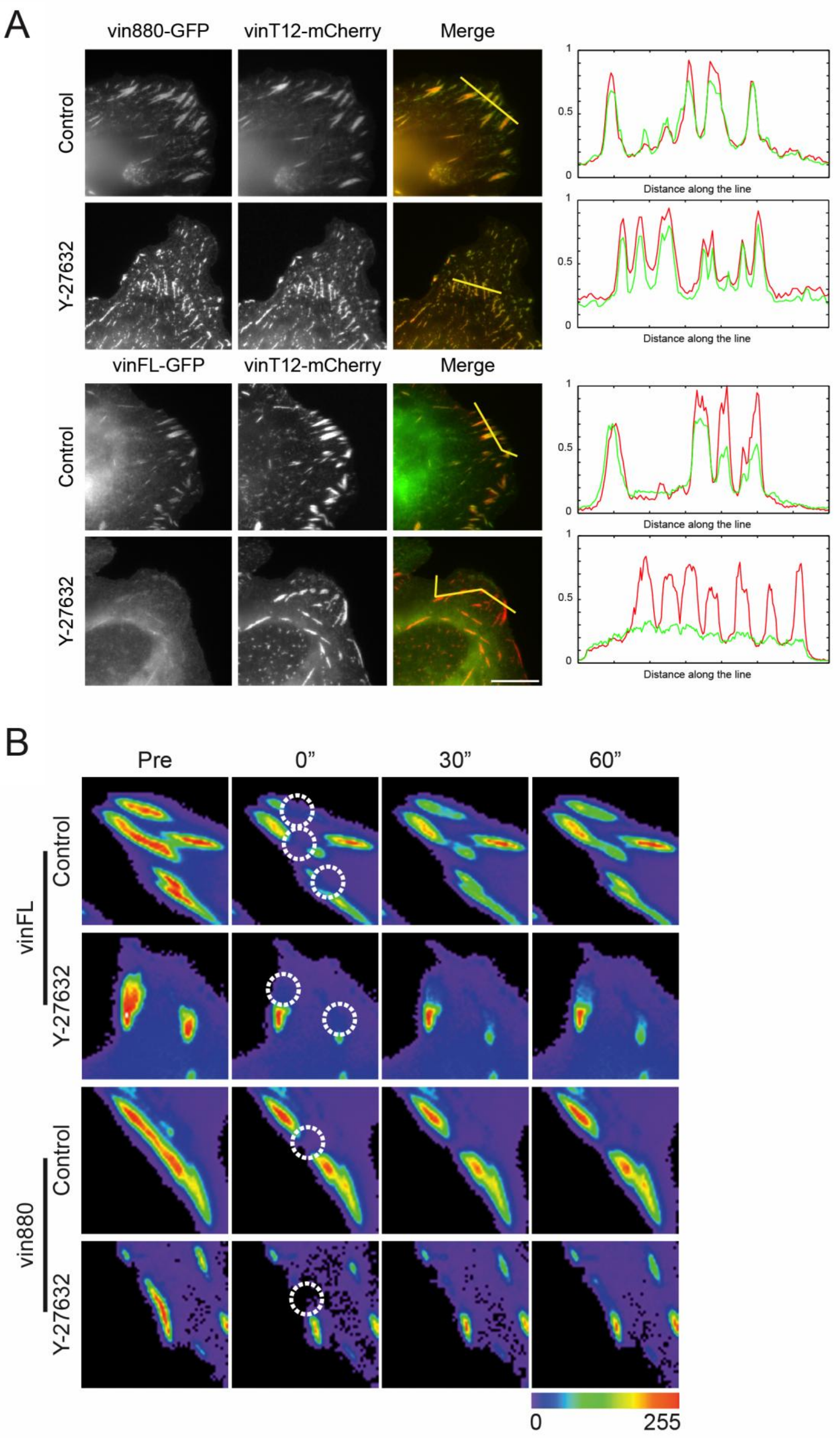

A
vin880-GFP
vinT12-mCherry
Merge
Control
Y-27632
vinFL-GFP
vinT12-mCherry
Merge
Control
Y-27632
1
0.5
0
Distance along the line
B
Pre
0”
30”
60”
vinFL
Control
Y-27632
vin880
Control
Y-27632
0
255

**Figure S3, Related to Figure 3. Vinculin Full-Length Leaves Stabilised FAs upon Y-27632 Treatment, and Subsequent Recruitment Is Perturbed**

(A) U2-OS cells co-expressing vin880 with vinT12 or vinFL with vinT12. Note that after the Y-27632 treatment, the FA stabilising vin880 and vinT12 couple still co-localise in FAs. In contrast, vinFL leaves vinT12-stabilised adhesions upon the drug treatment. Line profiles are plotted according to the intensities of staining along the yellow line. Scale bar, 5 μm.

(B) Representative FRAP time lapse panel of MEFvin$^{-/-}$ cells expressing vinFL-GFP or vin880-GFP treated with or without Y-27632. White circles indicate bleached regions of interest. Images were converted to an 8-bit rainbow RGB colour scale to clearly identify the fluorescence recovery. Note that the recovery of vinFL was perturbed after Y-27632 treatment.

# Figure S4

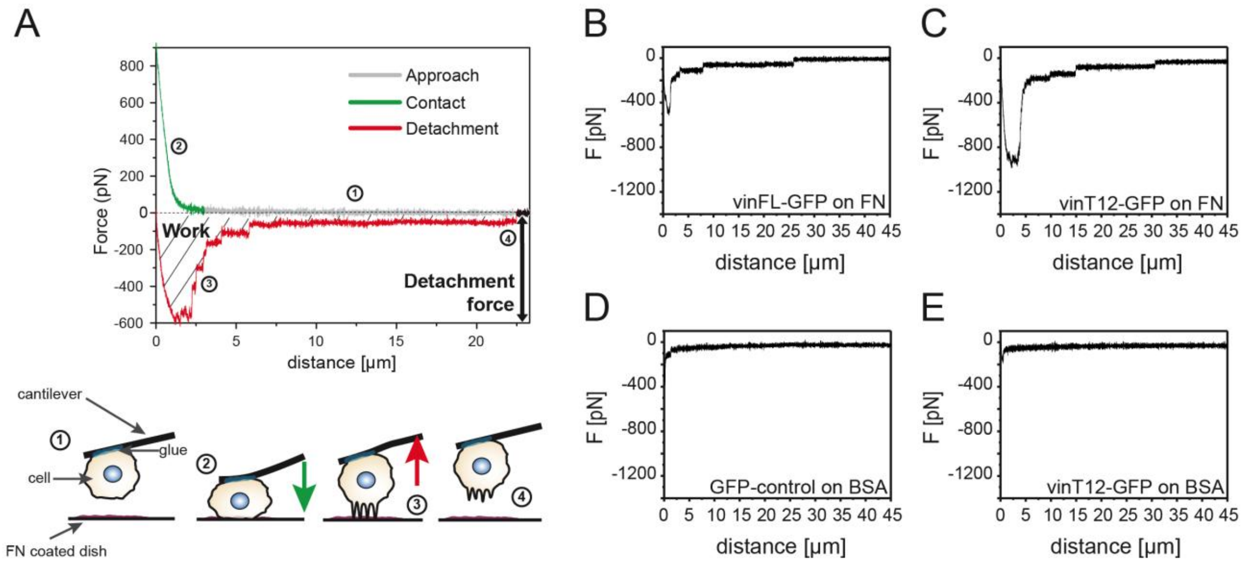


**Figure S4, Related to Figure 4. Adhesion of MEFvin$^{-/-}$ Cells from the Cantilever onto the FN-Coated Dish Is Integrin-Dependent**

(A) Schematic outlining individual steps involved in atomic force spectroscopy measurements that were used to quantify the detachment forces of cells from FN-coated glass bottom dishes. Cells were attached to the tip of the cantilever using polyphenolic glue (1) and pressed with a force of 1 nN for 5 seconds to the FN-coated surface (2) before the cantilever was retracted (3 and 4). The analysis of the retraction curve allows measurements of the maximal force and working energy required for detachment of cells.

(B–E) Example of force-distance curves obtained with AFM when measuring cell adhesion with a vinFL (B) or vinT12 (C) expressing cell. Note the larger area under the curve (i.e., total work) and maximum peak (i.e., maximum detachment force) in the vinT12 condition compared to vinFL. The same experiment performed over a BSA-coated glass bottom dish shows that cells do not attach to it (D and E).

# Figure S5

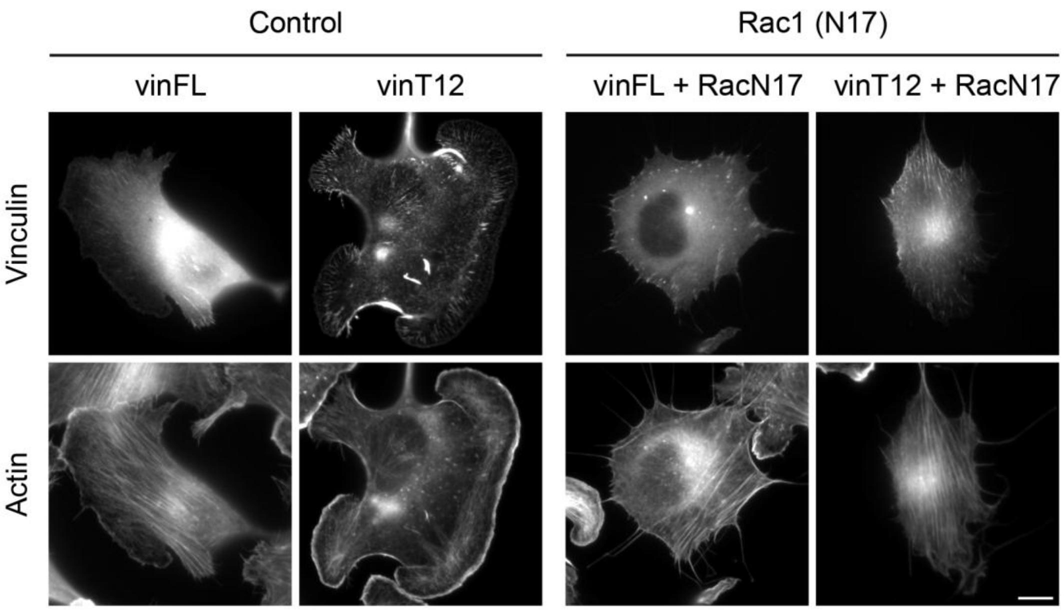


**Figure S5, Related to Figure 5. The Mislocalised Protrusive Activity in B16F10 Cells Expressing Vinculin Mutants Is Rac1 Driven**

The formation of multiple and/or branched lamellipodia in B16F10 cells expressing vinT12 is abolished by the co-expression of dominant negative Rac1N17. Scale bar, 5 μm.

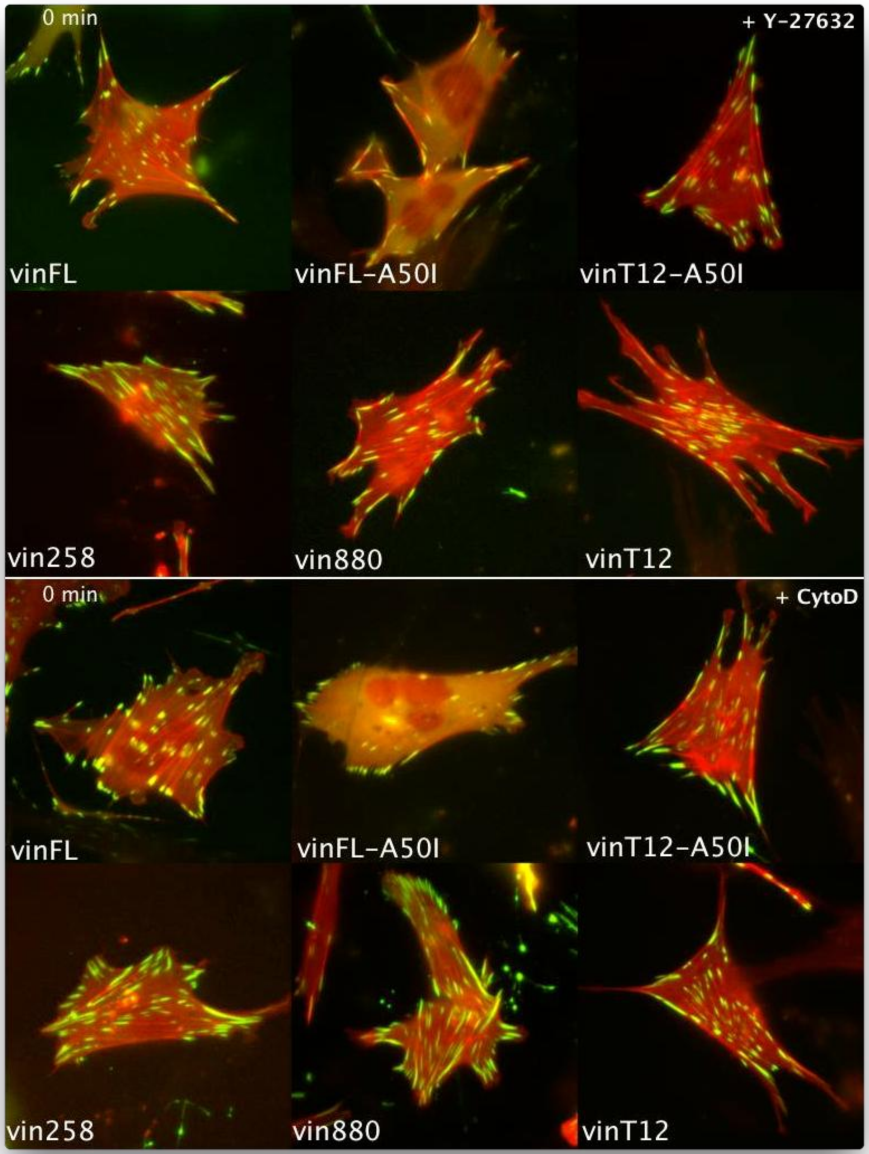


**Movie S1, Related to Figure 1. Recordings of FAs from MEFvin$^{-/-}$ Cells Expressing Indicated Constructs when Treated with the Rho-Kinase Inhibitor Y-27632, in the Top Panel, or the Actin-Disrupting Agent Cytochalasin D, in the Bottom Panel**
Note that only vin258, vin880 and vinT12 maintain the FAs stable throughout the tension-releasing treatment. An A50I mutation that reduces the vinculin-talin interaction strength abolishes the FA stabilising effect of these constructs. Video displays images taken every minute. Images are played back at 5 frames/s.

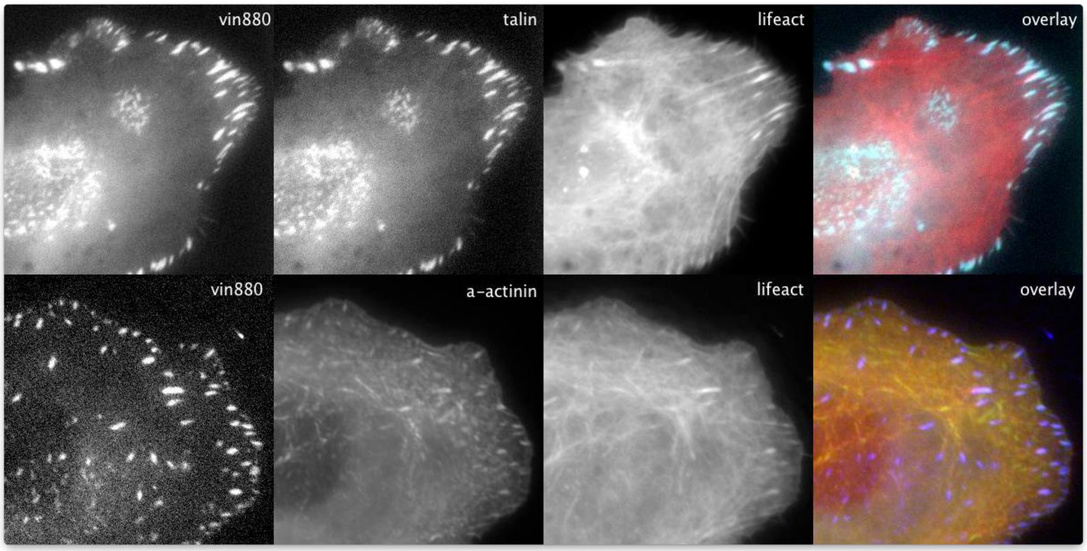


**Movie S2, Related to Figure 2. Time-Lapse Recording of a U2-OS Cell Coexpressing Talin-YFP, vin880-CFP, and LifeAct-mRFP or α-actinin-YFP, vin880-CFP, and LifeAct-mRFP and Treated with Cytochalasin D**
Note that both vin880 and talin remain stable, identically localising over the course of stress fibre disassembly. In contrast, α-actinin disappears from FAs and follows the actin positive structures in the cell. Video displays images taken every minute. Images are played back at 10 frames/s.

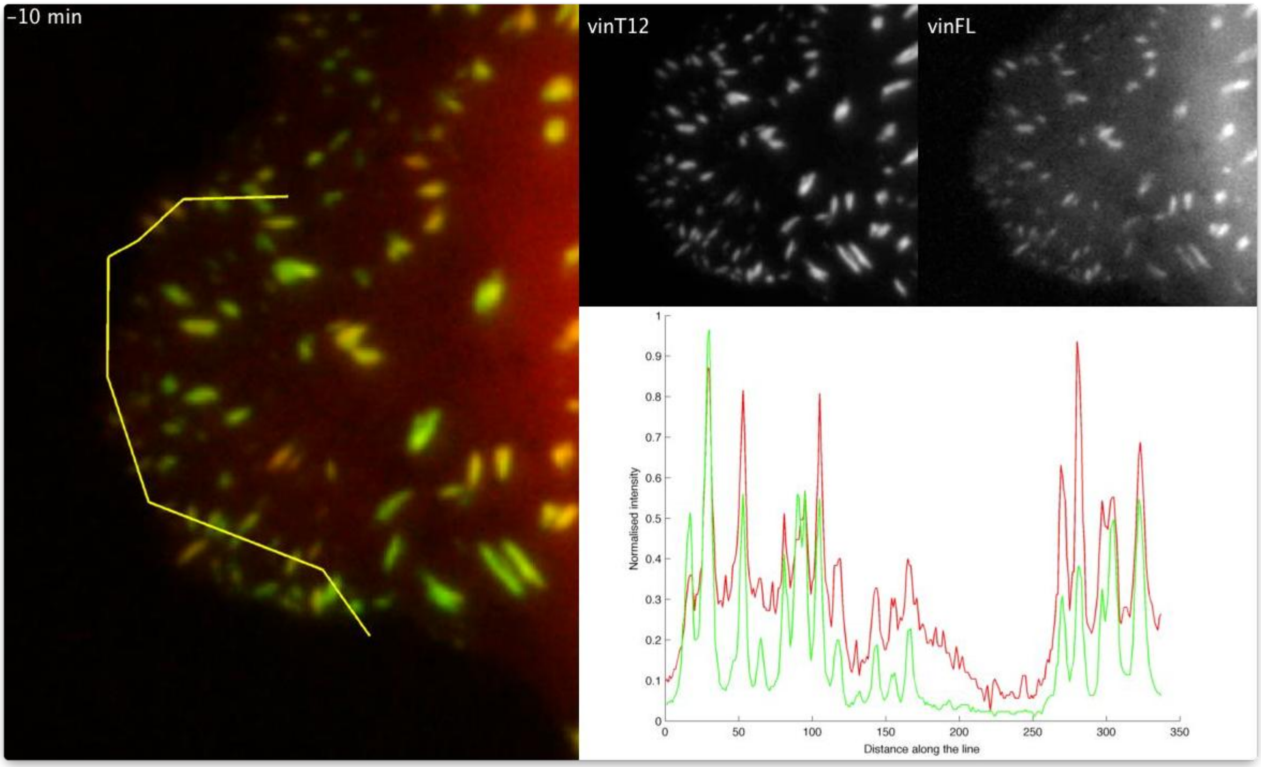


**Movie S3, Related to Figure 3. Time-Lapse Recording of U2-OS Cell Coexpressing vinFL and vinT12**

Cells are treated with 100 μM of Y-27632 at the beginning of the movie. Time is indicated at the top left in minutes. The red and green profiles reveal the normalised intensities of vinFL-mCherry and vinT12-GFP respectively along the yellow line drawn on the merged image. Images are played back at 10 frames/s.

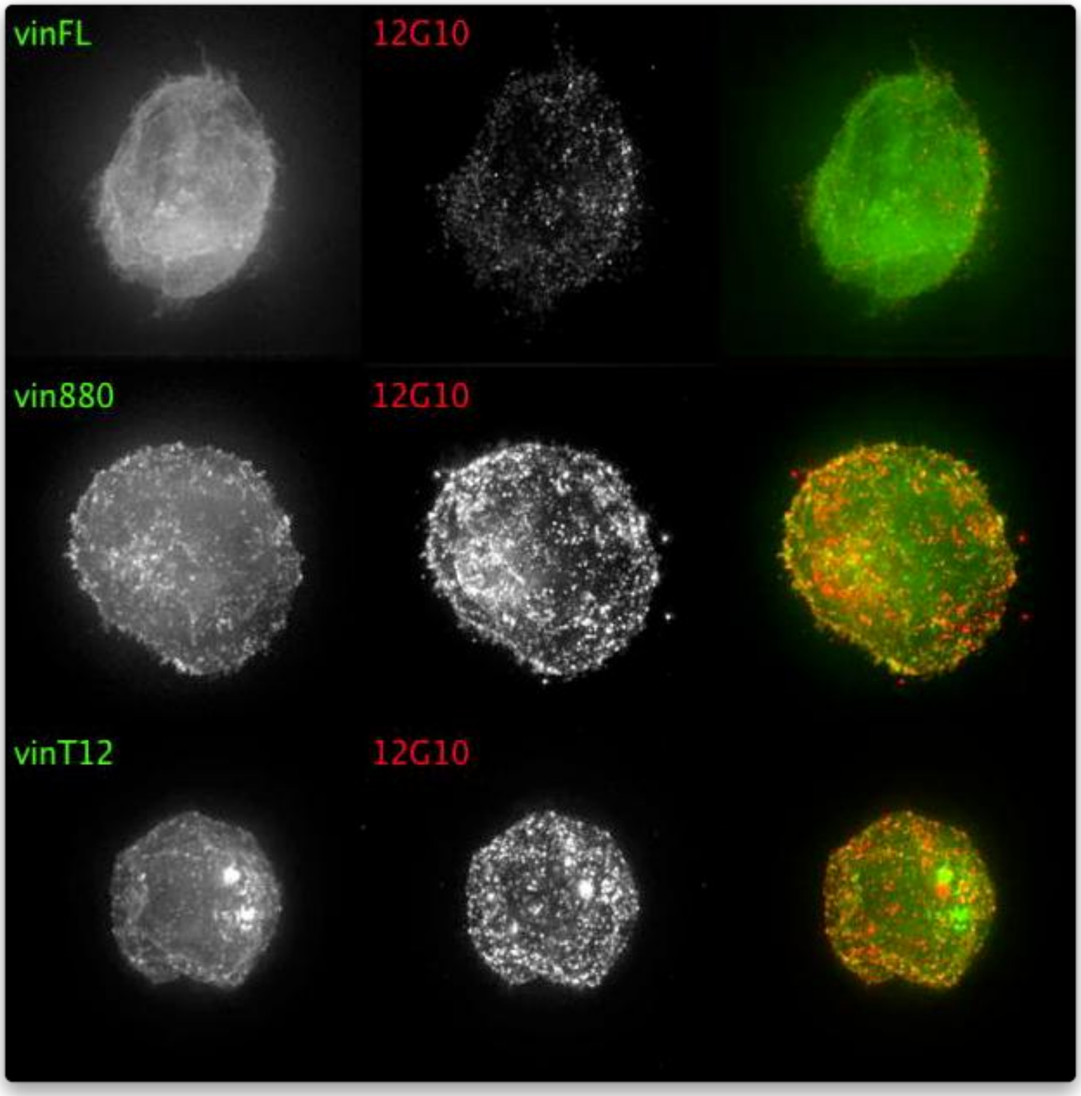


**Movie S4, Related to Figure 4. Three-Dimensional Reconstruction of U2-OS Cells Expressing the Indicated Vinculin Constructs**

Cells were co-stained with 12G10 antibody to detect activated β1-integrins. Only cells expressing the FA stabilising vinculin forms vin880 or vinT12 were able to maintain clusters positive for active β1-integrins in round cells in the absence of intracellular tension. All immunofluorescence images were captured with the same exposure times for direct comparison and quantification of immunostainings.

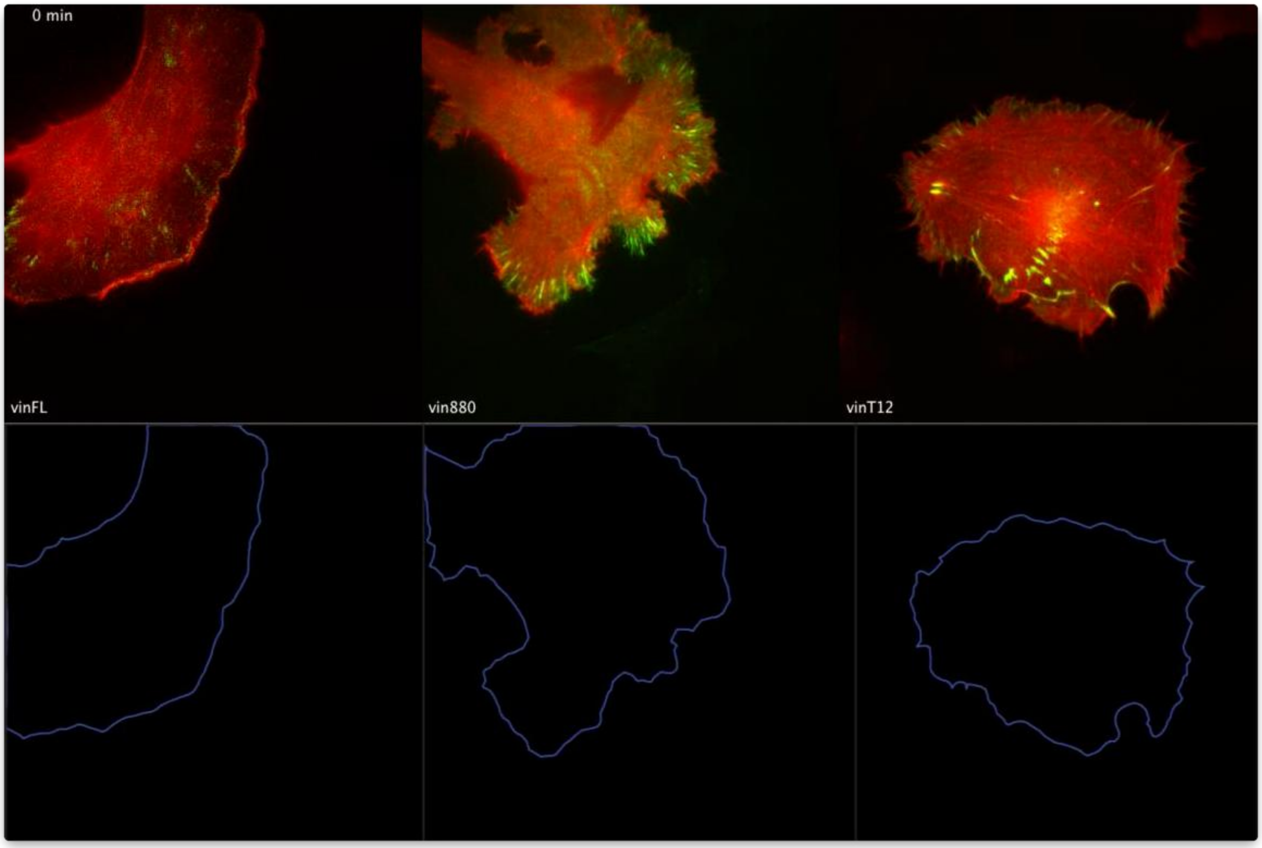


**Movie S5, Related to Figure 5. Recordings of FAs and Actin Dynamics from B16F10 Cells Coexpressing Indicated Vinculin Construct and LifeAct-mRFP when Plated on Laminin**

Cells expressing active vinculin constructs (vin880 or vinT12) exhibit protrusive activity all around the cell while the cell expressing vinFL displays space- and time-restricted protrusive activity. The protrusive activity is highlighted by the colour-coded cell shape outlines during cell recordings in time. Video displays images taken every minute. Images are played back at 10 frames/s.

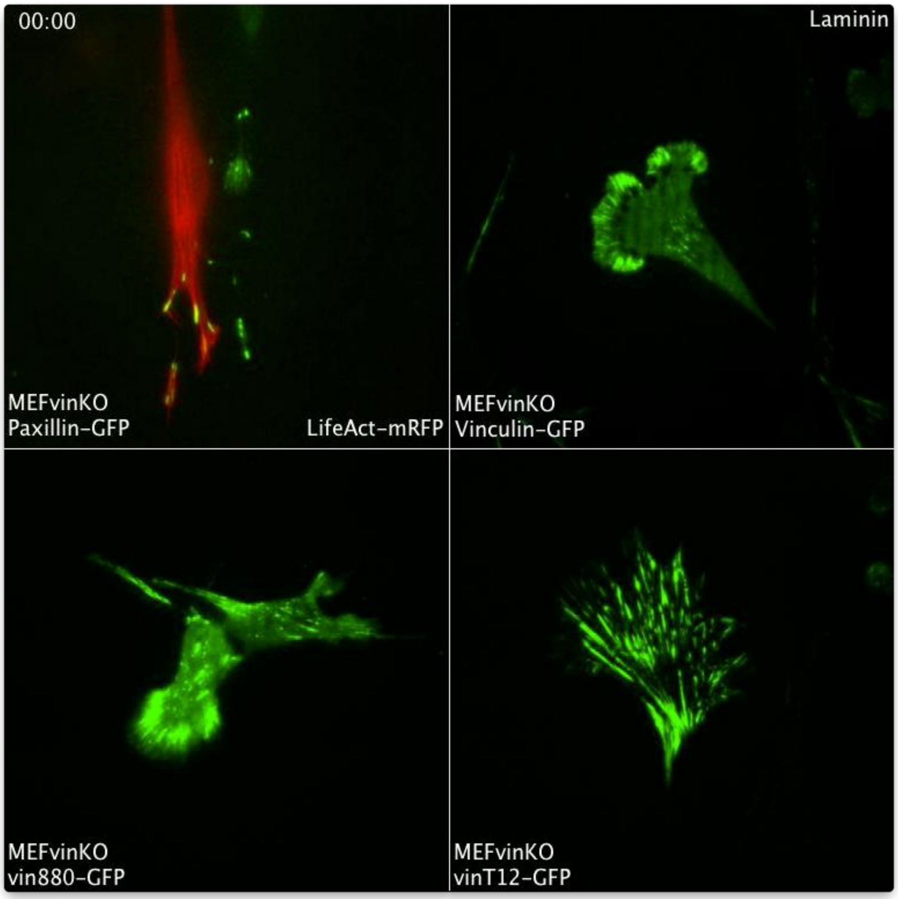


**Movie S6, Related to Figure 5. MEFvin$^{-/-}$ Change Their Migration Mode after Re-expression of Vinculin**

MEFvin$^{-/-}$ cells expressing the indicated constructs are plated on 5 μg/ml of laminin and imaged using TIRF microscopy (or epifluorescence microscopy for LifeAct-mRFP only). Re-expression of full-length vinculin induces the rescue of polarised migratory behaviour, which is strikingly different from MEFvin$^{-/-}$ cells and characterised by an increase in size and number of FAs. The expression of any active vinculin construct (vin880 or vinT12) leads to a more static phenotype where cells protrude in every direction but fails to migrate effectively. This change of phenotype is comparable to what was characterised for B16F10 cells (see figure 5C). Images are played back at 10 frames/s.

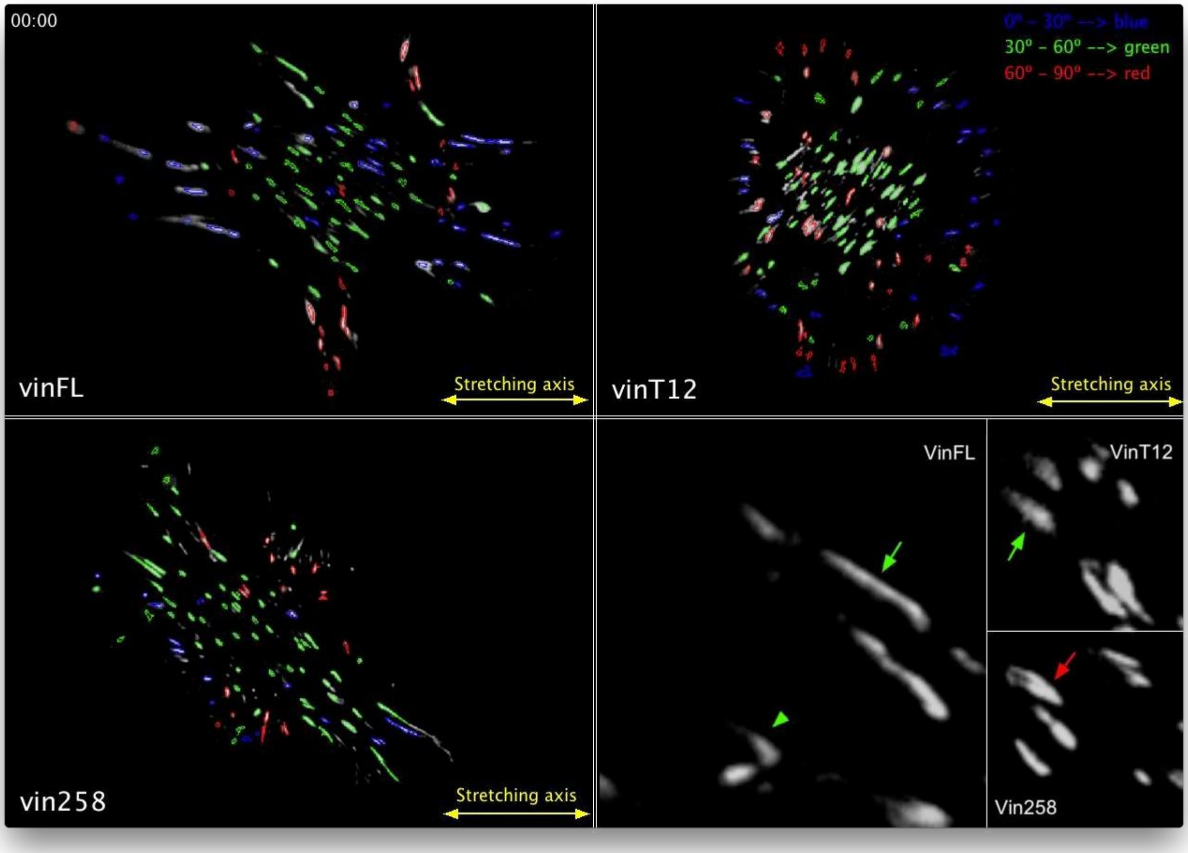


**Movie S7, Related to Figure 6. Time-Lapse Recording of MEFvin$^{-/-}$ Cells Expressing the Indicated Vinculin Construct during Substrate Stretching**

Cells are expressing the GFP-tagged vinculin construct as indicated. The perimeter of each FA is a colour-coded representation of the FAs based on their orientation at different time points during cyclic stretching. The orientation angle is determined as the angle between the main axis of each FA and the stretching axis (indicated with the yellow double arrow). Briefly, cells expressing vinT12 reorganise their FAs to become perpendicular to the stretching axis faster than cells expressing vinFL. FAs in cells expressing vin258 that lacks the actin-binding site in the vinculin tail fail to reorientate during the stretching time course. The bottom right panel depicts typical examples of FA dynamics during the time course of the experiment. The green arrows point towards FAs whose orientation changes immediately under tension; the arrowhead points to a FA, which is first formed parallel to stretching forces, but then switches orientation after an initial phase of protrusion. The red arrow points towards a typical FA in a cell expressing vin258, which does not change its reorientation after application of stretch. Images are played back at 2 frames/s.

## Supplemental Experimental Procedures

### Antibodies, Reagents, and Plasmids

The various antibodies against β1 integrin subunits (clones K20 and 12G10) were a gift from S.E. Craig and M.J. Humphries (Wellcome Trust Centre, Faculty of Life Sciences, University of Manchester, Manchester, UK) and described in [1]. Antibody against talin (clone TA205) and paxillin (clone 349) were purchased from Millipore while antibodies against paxillin pY118 (clone 44-722G) and FAK pY397 (clone 141-9) were purchased from Life Technologies. Secondary antibodies conjugated to DyLight-488, -594 or -649 were all from Jackson ImmunoResearch Laboratories and tested to avoid interspecies cross reactivity. Texas-Red-X-conjugated phalloidin and coumarin-conjugated phallacidin were from Life Technologies. Bovine plasma fibronectin, mouse basement membrane laminin (LM-111 chain composition: α1β1γ1), bovine serum albumin (BSA), ROCK inhibitor Y-27632, blebbistatin and cytochalasin D were from Sigma-Aldrich. The following constructs originated from the indicated labs: tensin, VASP, ILK, parvin and paxillin constructs from B. Geiger (Weizmann Institute of Science, Rehovot, Israel); GFP-vinT12 from S.W. Craig (John Hopkins School of Medicine, Baltimore, MD); GFP-α-actinin from C. Otey (University of North Carolina at Chapel Hill, Chapel Hill, NC); FAK, p130Cas, was from R. Horwitz (University of Virginia, Charlottesville, VA); talin constructs from K. Yamada and K. Matsumoto (National Institute of Dental and Craniofacial Research, Bethesda, MD); LifeAct-mRFP from R. Wedlich-Soeldner (Max Planck Institute of Biochemistry, Martinsried, Germany). α- and β-vinexin constructs from N. Kioka (Kyoto University, Kyoto, Japan) and ponsin from R. Tikkanen (Universität Gießen, Gießen, Germany). The Arp2/3-C2 plasmid from K. Rottner (Helmholtz-Zentrum für Infektionsforschung, Braunschweig, Germany); zyxin plasmid from M. Beckerle (University of Utah, Salt Lake City, UT).

### Transfection

For transient transfections, Lipofectamine and Plus reagents were used with B16F10 and MEFvin$^{-/-}$ cell lines, and Lipofectamine 2000 (Life Technologies) with U2-OS cells, according to the manufacturer's instructions, on cells in 6-well dishes, seeded 24 hours before transfection and re-plated 4 hours after transfection.

### Immunofluorescence, Live Imaging of FAs Disassembly, and Image Processing

For immunofluorescence, 13 mm coverslips or glass bottom dishes (MatTek Corporation) were coated for 4h at room temperature with 10 μg/ml fibronectin diluted in PBS and rinsed twice with PBS. After transfection, cells were plated in complete DMEM and allowed to spread for 16 h. For the actin-disruption experiments, cells were treated for 1 h at 37°C with 100 μM of Y-27632 diluted in water or with 2 μM cytochalasin D diluted in DMSO. Control conditions were treated with similar concentration of the carrier agent only. Cells were fixed with 4% (wt/vol) PFA and permeabilised with 0.5% (vol/vol) Triton X-100 (both from Sigma-Aldrich) diluted in PBS. Cells were subsequently incubated for 1 hour with primary antibodies directed against indicated proteins. After three washes with PBS, cells were incubated in the presence of secondary antibody. Staining for actin was performed together with the secondary antibody for 1 hour when required. After three washes, coverslips were mounted on slides using Mowiol

containing DABCO (Sigma-Aldrich). Samples were observed on a Deltavision RT microscope (Applied Precision) using a 100x NA 1.35 Uplan Apo objective (Zeiss) and 86000v2 or 86006 filter sets (Chroma). Images were collected using a Coolsnap HQ CCD (Photometrics) camera, converted and analysed using Fiji software (v1.45m to 1.46j). Raw images were subjected to signal re-scaling using linear transformation only for display purposes in the figures. For calculation of the Pearson's correlation coefficient, multi-channel stacks were background-subtracted using a two-dimensional high-pass filter [2], a region of interest was drawn and a threshold was set to restrict analysis to peripheral FAs. The coefficient was measured using the Intensity Correlation Analysis plugin for Fiji [3] and the subsequent plotting step was performed using MATLAB (version R2012a; MathWorks). A minimum of fifteen cells were analysed per condition.

For live imaging of FA disassembly, MEFvin$^{-/-}$ cells expressing GFP-tagged vinculin constructs and LifeAct-mRFP were seeded on fibronectin-coated glass bottom dishes (10 μg/ml) and allowed to spread for 16 hours in complete medium before capturing time-lapse images. Images were collected on a Nikon TE2000 microscope, equipped with a Perfect Focus System to eliminate focus drift, using the 100x NA 1.49 Apo TIRF objective. The images were then collected through MetaMorph software (Molecular Devices) using a Cascade 512B EM CCD camera (Photometrics) at 1 min intervals for up to 2 hours. Custom Fiji macros and MATLAB scripts were used for normalization of FA number and area fraction. Briefly, raw images were assembled into stacks and the edge of each cell was manually drawn using the LifeAct-RFP signal to obtain the total cell area. The vinculin-GFP signal has been processed using a two-dimensional high-pass filter and a rolling ball noise subtraction to create a binary mask of the FAs. Using this mask and the raw images, the intensity of the signal within the FAs and the area fraction (*i.e.* percentage of the total cell area occupied by FAs) was calculated per frame. Then, for each cell separately, the intensity and number of FAs at the initial time point of the recording (*i.e.* 5 or 10 minutes before the use of the drug) was set to be 100%. Using 10 to 12 cells originated from 2 independent experiments, the average curve and S.E.M. was calculated to produce figures 1C and S1B.

**FRAP Experiments**

MEFvin$^{-/-}$ cells transfected with GFP-vinFL or GFP-vin880 were plated onto fibronectin coated glass bottom dishes (10 μg/ml). DME medium was replaced with Ham's F-12 medium supplemented with 25 mM HEPES buffer, 1% penicillin/streptomycin and 1% L-glutamine one hour prior to imaging and left to equilibrate in a pre-warmed environment at 37$^{o}$C and 5% $CO_2$. FRAP studies were performed using a Deltavision RT microscope equipped with a 100x NA 1.40 UPlan Apo oil immersion objective (Zeiss). FRAP laser beam diameter size was 1 μm and was held for 0.075 s per region of interest, to bleach. Images were recorded every 10 s for 5 min according to previously published methods [4]. Normalised fluorescence intensities were obtained using SoftWorx FRAP analysis software (Applied Precision). Data were imported into MATLAB and fitted with single exponential curve fits according to published FRAP models [5]. A custom MATLAB script was used to analyse and fit the data. A rainbow RGB look-up table on an 8-bit colour intensity scale was applied to the images to clearly indicate the changes in fluorescence intensities.

**Round Cells Assay**

For the immunolabelling of non-spread cells, transfected U2-OS cells were detached using trypsin/EDTA, washed once in PBS/EDTA and twice in PBS before being re-suspended in PBS with or without 100 μM of Y-27632 for 20 minutes. They were then seeded on poly-D-lysine coated glass bottom dishes at room temperature for less than 10 minutes before being fixed and processed as described above. Three dimensional image stacks were taken using a Deltavision RT microscope with a z-spacing of 0.1 μm before being deconvoluted (aggressive mode, 10 pass) using SoftWorx software. All images were taken with constant exposure time between all the conditions of the same antibody staining, thus allowing for quantitative comparison. Quantification was performed on the raw data after background subtraction and mathematical summing of all z-slices using custom Fiji macros. Subsequently, data were assembled into a 3D volume using Fiji and subjected to signal re-scaling using linear transformation for display in the figures and supplemental movies.

**Cell Migration Assay and Live Imaging of Cell Protrusions**

Transfected B16F10 melanoma cells were seeded on laminin-coated glass bottom six well plates (2 μg/ml) and allowed to spread for 16 h in serum-free medium before capturing time-lapse images. Images were acquired on a Nikon TE2000 microscope equipped with a Perfect Focus System to eliminate focus drift, using a 40x objective in stitching mode to acquire 3x3 field arrays of images. The images were collected through the Elements software using a Nikon DQC-FS EM-CCD camera at 30 min intervals for 24 hours. Migration paths of transfected, non-dividing, non-clustered cells were tracked using the MTrackJ plugin for Fiji [6] and migration plots were reconstructed using the Chemotaxis and Migration tool plugin for Fiji (Ibidi) and analysed using SPSS Statistics (v 20, IBM). For the tracking of polarised cell migration at higher magnification, images were acquired using the same imaging system but with a 100x NA 1.49 Apo TIRF objective. A 585 nm Precise LED fluorescent light source was used for the LifeAct-mRFP signal and a 488 nm argon laser line operating in TIRF illumination mode was used for vinculin GFP-tagged signal. Raw movies were further post-processed in Fiji. Briefly, contrast was adjusted using an empirically determined linear transformation and colour-merged movies were created after correction for stage drift using StackReg plugin for Fiji [7]. The detection of the outline of the cells over time was performed using the QuimP11 toolbox, kindly provided by Till Bretschneider and Richard Tyson (University of Warwick, Coventry, UK, [8]).

**Imaging of Rac Activity Using a FRET Reporter and Dominant Negative Rac1N17**

B16F10 melanoma cells were transfected similarly as above to express mCherry-tagged vinculin constructs and Raichu-Rac FRET sensor [9] and plated on laminin for 16 hours without serum. After fixation with paraformaldehyde, cells were imaged with the same Deltavision RT microscope previously described using the following filters settings: CFP (excitation, EX) / CFP (emission, EM), CFP (EX) / YFP (EM), YFP (EX) / YFP (EM) and mCherry (EX) / mCherry (EM). After background subtraction, a ratio image of CFP:YFP to CFP:CFP was calculated and plotted using a rainbow-coloured intensity scale. Image processing was performed using the same custom MATLAB script for background subtraction, normalisation and display. For the dominant negative

Rac1 expression assays, cells were transfected with Rac1N17 [10] and GFP-tagged vinculin constructs and stained with Texas Red X phalloidin.

**Single-Cell Spectroscopy**

Force measurements were performed with an atomic force microscope (AFM) (Nanowizard, CellHesion 200; JPK Instruments) installed on a fluorescence microscope (Axiovert 200M; Zeiss), on MEFvin$^{-/-}$ transfected with vinFL, vin880, vinT12 or an empty vector (all N-terminus GFP-tagged) 16 hours prior to the measurements. Glass bottom dishes (poly-D-Lysine coated; World Precision Instruments) were partly (one half of the chamber) coated with fibronectin (10 μg/ml), and saturated with a 1% BSA solution (in PBS) at 4°C. Chambers were rinsed with PBS and measurements were carried out in serum-free HEPES buffered medium. Tip-less AFM cantilevers (Arrow TL1, k = 0.03 N/m; NanoWorld) were coated with a polyphenolic protein glue [11] (Cell-Tak, BD). Cantilevers were calibrated in the medium-filled chambers using the thermal noise method [12]. The free end of the cantilever was brought into contact with a transfected cell in the non-coated half of the chamber, by approaching the cell from above with a speed of 5 μm/s. The cantilever was held in contact with the cell for 20 seconds at a loading force of 2 nN, after which the cantilever with the attached cell was brought up from the surface and left for 20 minutes to equilibrate. Subsequently, cells were brought into contact with FN by approaching the substrate at a speed of 5 μm/s until a loading force of 1 nN was reached. After a contact time of 5 seconds, cells were detached from the substrate with a retraction speed of 5 μm/s. This procedure was repeated twice in between 20 second pauses at a retraction distance of 90 μm. In total, four different FN areas were probed per cell. Force-distance curves consist of two parts (figure 4A): the approach and attachment to the FN coated substrate (top curve) and the retraction and detachment from the substrate (bottom curve). Two parameters were used for the characterization of the strength of the cell-FN adhesions: the detachment force, which corresponds to the maximum downward force on the cantilever and the work of detachment, which is the integral of the retraction force-distance curve. All data were analysed using the built-in JPK software. In the cases that there was no complete detachment between cells and FN at a pulling distance of 90 μm, the data was discarded. To prevent underestimation of these parameters, we took for each cell the average value of the detachment force and work, and averaged those over all the cells for each condition. All measurements were recorded at 20-22°C, to avoid cell spreading on the cantilevers.

**Cell-Stretching Experiments**

Stretching experiments were performed as described elsewhere [13]. Briefly, transfected MEFvin$^{-/-}$ were plated on fibronectin-coated poly(dimethylsiloxane) (Corning Sylgard) elastomeric membranes in carbonate-free Ham's F-12 media supplemented with L-glutamine, 2% FCS, 25 mM HEPES and penicillin-streptomycin. Time-lapse movies of at least five cells per experimental condition were taken every 10 minutes for 3 hours. Parameters for cyclic stretching were set to 1 Hz and 10% of linear stretch amplitude. Raw movies were processed to identify FAs and measure their orientation with respect to their initial angle to the stretching axis using custom MATLAB routines. Briefly, signal contrast was enhanced, background was subtracted using both FFT

bandpass filtering and a rolling ball method before the images were smoothed. Individual FAs were detected and fitted into an ellipse to register the angle between the orientation of the long axis of the FA and the stretching axis using a custom MATLAB routine. Angle values varied between 0° (parallel to the stretching axis) and 90° (perpendicular to the stretching axis) and were plotted back onto the images to allow manual tracking of FAs. Results were formatted into heatmaps where only FAs that did not appear, merge or disappear during the first 90 minutes of the stretching process.

## Supplemental References


1. Byron, A., Humphries, J.D., Askari, J.A., Craig, S.E., Mould, A.P., and Humphries, M.J. (2009). Anti-integrin monoclonal antibodies. J. Cell. Sci. *122*, 4009-4011.
2. Zamir, E., Katz, B.Z., Aota, S., Yamada, K.M., Geiger, B., and Kam, Z. (1999). Molecular diversity of cell-matrix adhesions. J. Cell. Sci. *112 ( Pt 11)*, 1655-1669.
3. Li, Q., Lau, A., Morris, T.J., Guo, L., Fordyce, C.B., and Stanley, E.F. (2004). A syntaxin 1, Galpha(o), and N-type calcium channel complex at a presynaptic nerve terminal: analysis by quantitative immunocolocalization. J. Neurosci. *24*, 4070-4081.
4. Carisey, A., Stroud, M., Tsang, R., and Ballestrem, C. (2011). Fluorescence Recovery After Photobleaching. Methods Mol. Biol. *769*, 387-402.
5. Sprague, B.L., Pego, R.L., Stavreva, D.A., and Mcnally, J.G. (2004). Analysis of binding reactions by fluorescence recovery after photobleaching. Biophys. J. *86*, 3473-3495.
6. Meijering, E., Dzyubachyk, O., and Smal, I. (2012). Methods for cell and particle tracking. Meth. Enzymol. *504*, 183-200.
7. Thevenaz, P., Ruttimann, U.E., and Unser, M. (1998). A pyramid approach to subpixel registration based on intensity. IEEE Trans Image Process *7*, 27-41.
8. Tyson, R.A., Epstein, D.B.A., Anderson, K.I., and Bretschneider, T. (2010). High Resolution Tracking of Cell Membrane Dynamics in Moving Cells: an Electrifying Approach. Math Model Nat Pheno *5*, 34-55.
9. Itoh, R.E., Kurokawa, K., Ohba, Y., Yoshizaki, H., Mochizuki, N., and Matsuda, M. (2002). Activation of rac and cdc42 video imaged by fluorescent resonance energy transfer-based single-molecule probes in the membrane of living cells. Mol. Cell. Biol. *22*, 6582-6591.
10. Ridley, A.J., Paterson, H.F., Johnston, C.L., Diekmann, D., and Hall, A. (1992). The small GTP-binding protein rac regulates growth factor-induced membrane ruffling. Cell *70*, 401-410.
11. Waite, J.H., and Tanzer, M.L. (1981). Polyphenolic Substance of Mytilus edulis: Novel Adhesive Containing L-Dopa and Hydroxyproline. Science *212*, 1038-1040.
12. Hutter, J.L., and Bechhoefer, J. (1993). Calibration of Atomic-Force Microscope Tips. Rev Sci Instrum *64*, 1868-1873.
13. Goldyn, A.M., Rioja, B.A., Spatz, J.P., Ballestrem, C., and Kemkemer, R. (2009). Force-induced cell polarisation is linked to RhoA-driven microtubule-independent focal-adhesion sliding. J. Cell. Sci. *122*, 3644-3651.
