# Supplementary figures and images for: Vinculin Regulates the Recruitment and Release of Core Focal Adhesion Proteins in a Force-Dependent Manner
Source: Curr Biol. 2013 Feb 18;23(4):271–81. doi: 10.1016/j.cub.2013.01.009 (PMC3580286; doi:10.1016/j.cub.2013.01.009)

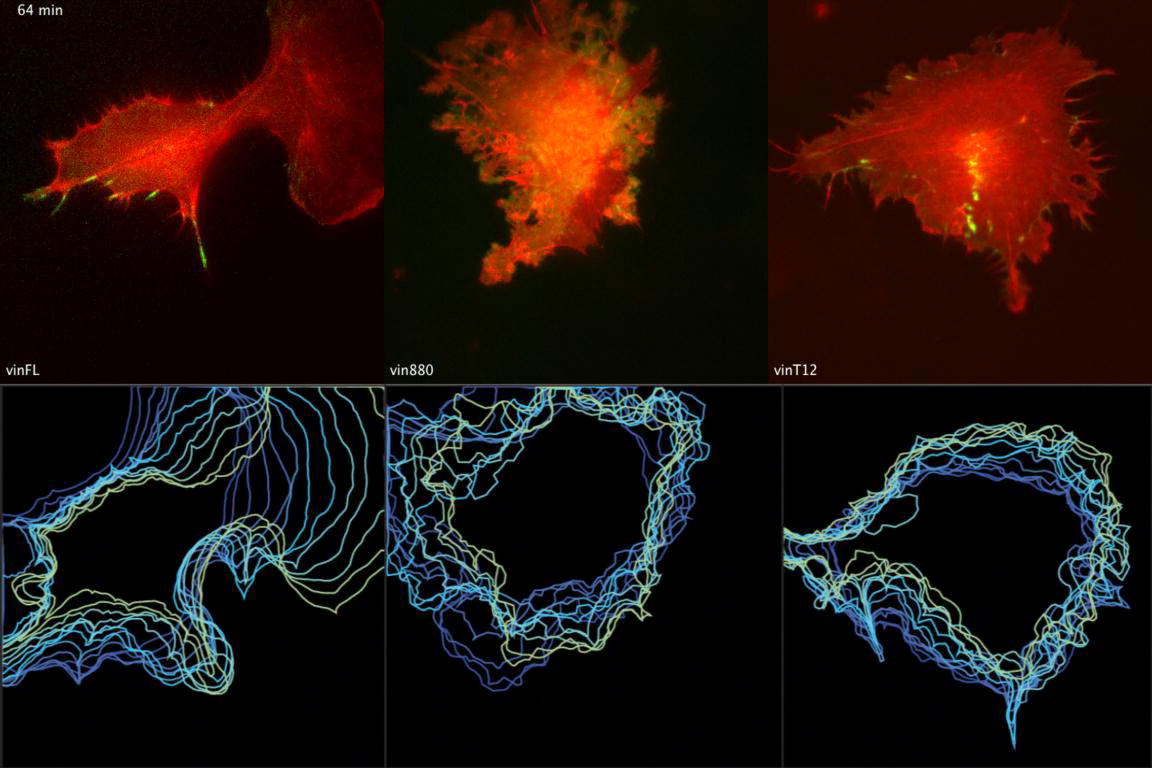

Supplement: Movie S5. Related to Figure 5. Recordings of FAs and Actin Dynamics from B16F10 Cells Coexpressing Indicated Vinculin Construct and LifeAct-mRFP when Plated on Laminin — Cells expressing active vinculin constructs (vin880 or vinT12) exhibit protrusive activity all around the cell, whereas the cell expressing vinFL displays space- and time-restricted protrusive activity. The protrusive activity is highlighted by the color-coded cell shape outlines during cell recordings in time. Video displays images taken every minute. Images are played back at 10 frames/s. [file mmc6.jpg]
